# Approaches to Decrease Hyperglycemia by Targeting Impaired Hepatic Glucose Homeostasis Using Medicinal Plants

**DOI:** 10.3389/fphar.2021.809994

**Published:** 2021-12-23

**Authors:** Gerardo Mata-Torres, Adolfo Andrade-Cetto, Fernanda Espinoza-Hernández

**Affiliations:** Laboratorio de Etnofarmacología, Departamento de Biología Celular, Facultad de Ciencias, Universidad Nacional Autónoma de México, Mexico City, Mexico

**Keywords:** medicinal plants, hyperglycemia, hepatic glucose output, insulin resistance, PTP-1B inhibitors, natural products

## Abstract

Liver plays a pivotal role in maintaining blood glucose levels through complex processes which involve the disposal, storage, and endogenous production of this carbohydrate. Insulin is the hormone responsible for regulating hepatic glucose production and glucose storage as glycogen, thus abnormalities in its function lead to hyperglycemia in obese or diabetic patients because of higher production rates and lower capacity to store glucose. In this context, two different but complementary therapeutic approaches can be highlighted to avoid the hyperglycemia generated by the hepatic insulin resistance: 1) enhancing insulin function by inhibiting the protein tyrosine phosphatase 1B, one of the main enzymes that disrupt the insulin signal, and 2) direct regulation of key enzymes involved in hepatic glucose production and glycogen synthesis/breakdown. It is recognized that medicinal plants are a valuable source of molecules with special properties and a wide range of scaffolds that can improve hepatic glucose metabolism. Some molecules, especially phenolic compounds and terpenoids, exhibit a powerful inhibitory capacity on protein tyrosine phosphatase 1B and decrease the expression or activity of the key enzymes involved in the gluconeogenic pathway, such as phosphoenolpyruvate carboxykinase or glucose 6-phosphatase. This review shed light on the progress made in the past 7 years in medicinal plants capable of improving hepatic glucose homeostasis through the two proposed approaches. We suggest that Coreopsis tinctoria, Lithocarpus polystachyus, and Panax ginseng can be good candidates for developing herbal medicines or phytomedicines that target inhibition of hepatic glucose output as they can modulate the activity of PTP-1B, the expression of gluconeogenic enzymes, and the glycogen content.

## Introduction

Diabetes mellitus (DM) is a chronic metabolic disease characterized by high blood sugar levels (hyperglycemia), caused by insulin malfunctioning, deficient insulin secretion, or both (Liu et al., 2019). Type 2 diabetes (T2D) is the most important type of DM due to its high worldwide prevalence (American Diabetes Association, 2021). It is characterized by insulin resistance, which is defined as a poor response of insulin-sensitive tissues to normal insulin concentration (Mlinar et al., 2007). The main cause of insulin resistance has been associated to an obesogenic environment in which large amounts of free fatty acids and adipokines are responsible for impairing insulin signaling by increasing serine phosphorylation that inhibits tyrosine phosphorylation of insulin receptor (IR) and insulin receptor substrates (IRSs) (DeFronzo et al., 2015). However, it has also been reported that protein tyrosine phosphatases (PTPs) could have a more important role since they are upregulated in insulin resistant states. Insulin action is negative regulated by PTPs, particularly the PTP-1B, because they promote the dephosphorylation of tyrosine residues of IR and IRSs (Saltiel and Kahn, 2001). When insulin signaling is impaired in liver by either insulin resistance or low insulin levels, the glucose storage and production is dysregulated, increasing the hepatic glucose output rates yielding hyperglycemia in diabetic patients.

Liver represents a crucial therapeutic target for treating hyperglycemia in T2D because hepatic glucose output is the pathophysiological abnormality that contributes the most to the hyperglycemic state in fasting and postprandial state as a consequence of hepatic insulin resistance (Sharabi et al., 2015). During the overnight fast (postabsorptive state), the liver of a normal person produces glucose at a rate of approximately 1.8–2 mg/kg. min. However, this rate increases around 0.5 mg/kg min in a patient with T2D, promoting a significant rise in the basal state of glucose production (Cersosimo et al., 2018). After food ingestion and the subsequent increase in insulin levels, the suppression of glucose production is slower in a diabetic patient, promoting an evident postprandial hyperglycemia due to the excess of glucose produced in addition to that from the exogenous source (Rizza, 2010).

Medicinal plants and natural products have shown to have numerous benefits on processes involved in glucose and lipid metabolism, leading to correct homeostasis imbalances that promote metabolic diseases such as T2D (Li J. et al., 2018; Xu L. et al., 2018; Saadeldeen et al., 2020). Unlike the classic “on-target” paradigm in pharmacology, namely a drug with a specific target, the polypharmacology approach, or the binding of a drug to more than one target, could be more effective against a disease as complex as T2D due to its multiple pathophysiological abnormalities (Reddy and Zhang, 2013). In this context, extract plants and phytochemicals isolated from medicinal plants exhibit multiple mechanisms of action on assorted metabolic targets that are involved in glucose homeostasis. Therefore, efforts have been made to describe all the beneficial effects on metabolism of these extracts and molecules in recent years.

The current review summarizes the medicinal plants reported from 2015 that can potentially decrease hyperglycemia resulting from imbalance in hepatic glucose metabolism by two different approaches: improving hepatic insulin resistance by inhibiting PTP-1B and decreasing hepatic glucose output by inhibiting rate-limiting enzymes involved in the storage and production of glucose.

## Methodology

**FIGURE 1:**
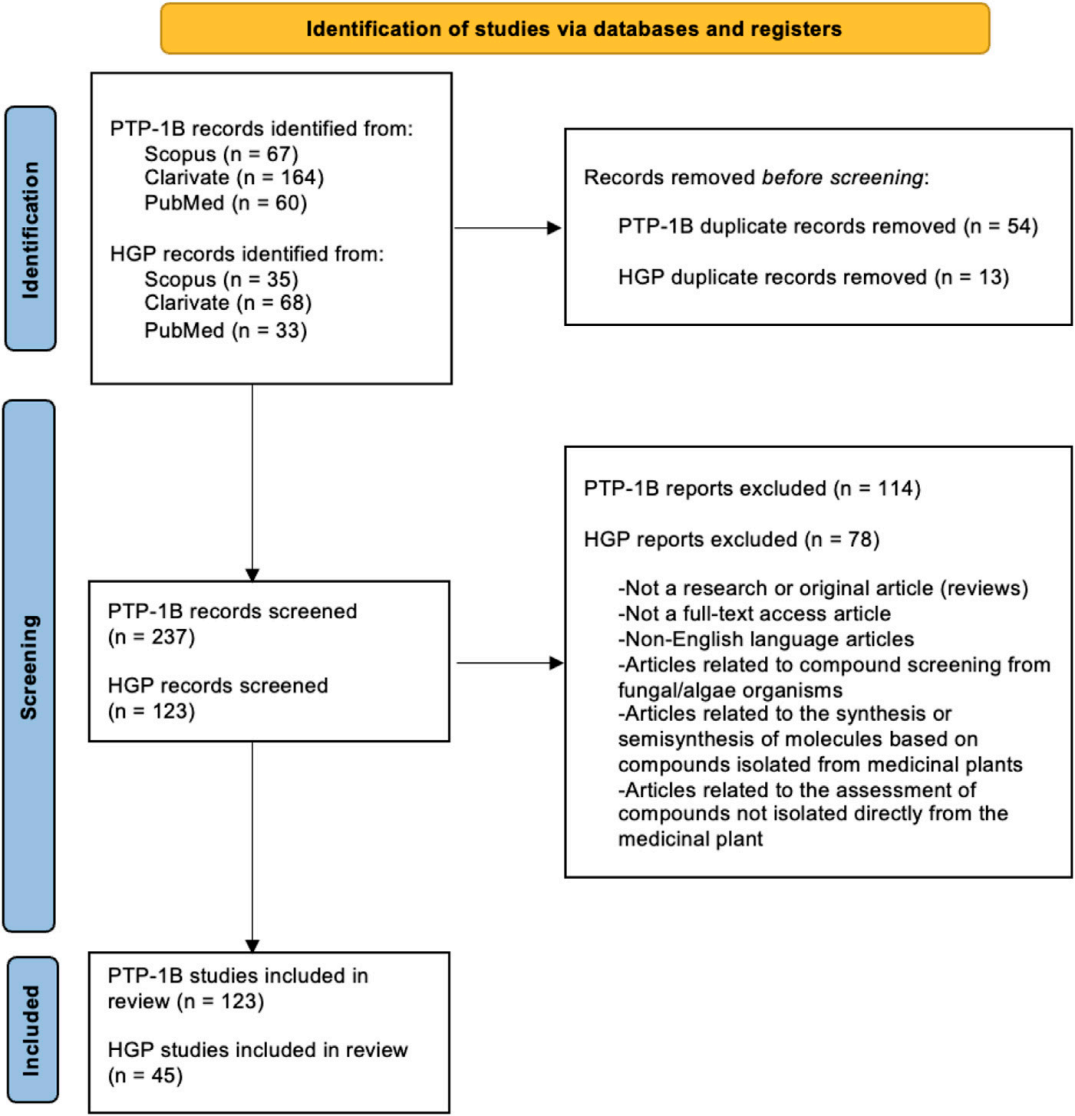
PRISMA flowchart. PTP-1B: protein tyrosine phosphatase 1B; HGP: hepatic glucose production.

Two separate searches were performed based on the Preferred Reporting Items for Systematic Review and Meta-Analysis (PRISMA) (Page et al., 2021) in the following databases: Scopus, Clarivate and PubMed (Figure 1). The first involved studies related to extracts or phytochemicals tested against the activity or expression of PTP-1B enzyme, while in the second, studies with extracts or phytochemicals with an effect on the glucose-producing pathways were sought. Only records related to the study of medicinal plants and their isolated compounds were considered.

## Therapeutic Approaches to Reduce Hyperglycemia Resulting From Impaired Hepatic Glucose Homeostasis

Each insulin-sensitive tissue presents abnormal characteristics that contribute to hyperglycemia in an insulin-resistant state. The underlying mechanisms that give rise to insulin resistance converge on deficient insulin signalling that limits the activation of factors involved in energy metabolism. In obesity and T2D, insulin resistance has been linked mainly to defects in the signalling pathway of phosphatidylinositol 3-kinase and protein kinase B (PI3K/Akt), particularly to the Akt2 isoform (Cusi et al., 2000; Krook et al., 2000).

In normal conditions, the insulin secreted by pancreatic β cell binds to its receptor in the target cell, activating the tyrosine kinase activity, which promotes the receptor autophosphorylation and the subsequent phosphorylation of IRSs, mainly IRS-1 and IRS-2, in tyrosine residues. Afterwards, the enzyme P13K is recruited and activated by IRS to convert phosphatidylinositol 4,5-bisphosphate (PIP2) from the plasma membrane to phosphatidylinositol 3,4,5-triphosphate (PIP3), which facilitates the phosphorylation and activation of Akt at two important sites: by phosphoinositide-dependent kinase 1 (PDK1) at residue Thr308 of the catalytic domain, and by mammalian target rapamycin complex 2 (mTORC2) at residue Ser473 of the regulatory domain (Schultze et al., 2012). Specifically in liver, the activated Akt enzyme is responsible for phosphorylating different factors that are involved in the regulation of processes such as glycogen synthesis, gluconeogenesis, and glycogenolysis, which are activated or inhibited under different nutritional circumstances (Dimitriadis et al., 2021).

**FIGURE 2:**
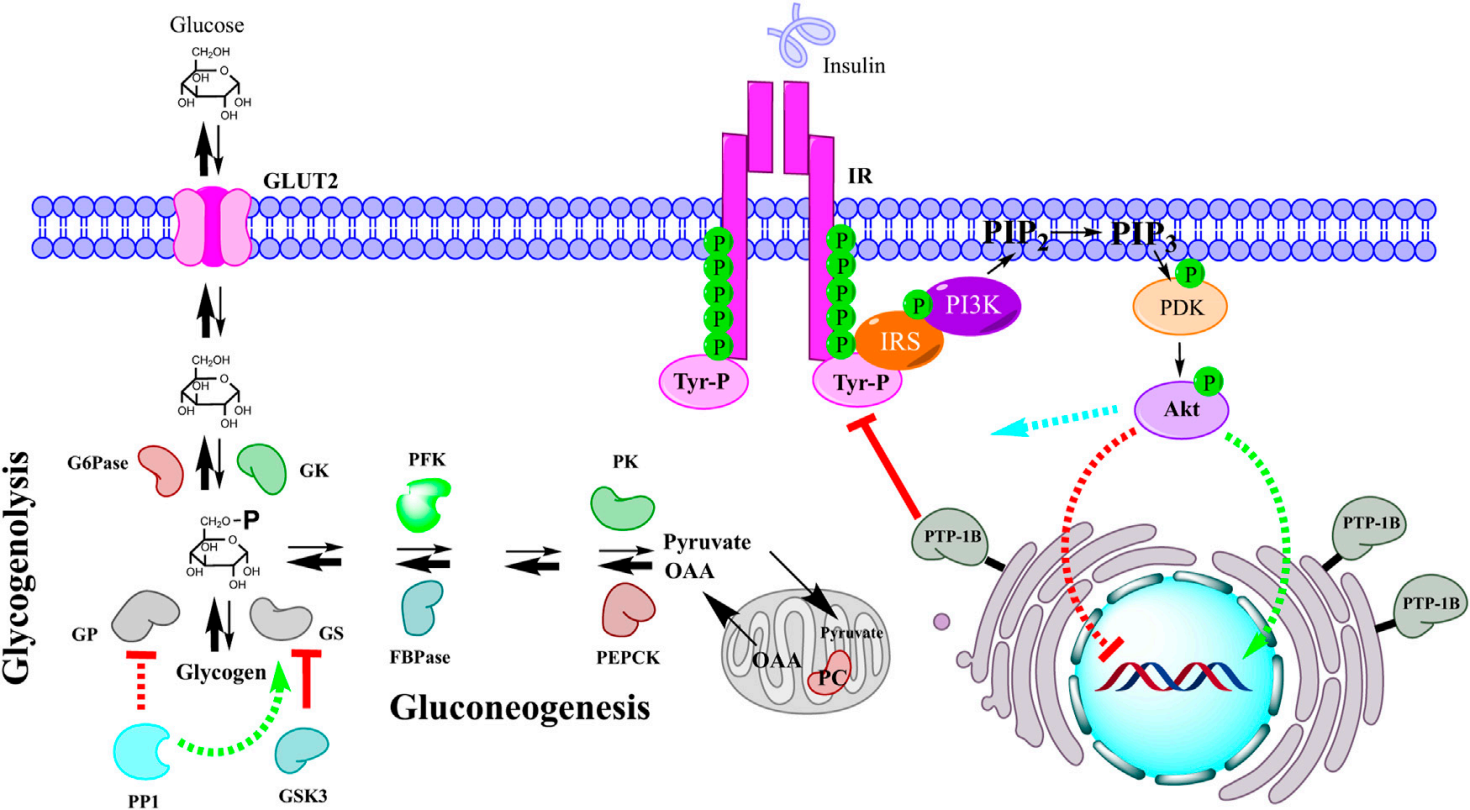
Impaired hepatic glucose homeostasis by insulin resistance. When insulin does not work properly either due to overexpression of PTP-1B or other factors, glucose production in liver is upregulated generating a hyperglycemic state. Both gluconeogenesis and glycogenolysis are enhanced due to poor insulin signaling, namely genetic expression of gluconeogenic enzymes is not repressed and enzymes related to glycogen metabolism are not adequately regulated. Akt functions: green color indicates positive regulation, red color indicates negative regulation, and blue color represents direct or indirect regulation by phosphorylation or allosterism. IR: insulin receptor; IRS: insulin receptor substrate; PI3K: phosphoinositide 3-kinase; PIP2: phosphatidylinositol 4,5-bisphosphate; PIP3: phosphatidylinositol 3,4,5-triphosphate; PDK: phosphoinositide-dependent kinase; Akt: protein kinase B; PTP-1B: protein tyrosine phosphatase 1B; PC: pyruvate carboxylase; OAA: oxalacetate; PEPCK: phosphoenolpyruvate carboxykinase; PK: pyruvate kinase; FBPase: fructose 1,6-bisphosphatase; PFK: phosphofructokinase; GS: glycogen synthase; GP: glycogen phosphorylase; PP1: protein phosphatase 1; GSK3: glycogen synthase kinase-3; GK: glucokinase; G6Pase: glucose 6-phosphatase; GLUT2: glucose transporter 2.

Due to hepatic insulin resistance, this hormone losses its ability to regulate glucose metabolism in liver, resulting in enhanced glucose output that contributes greatly to fasting and postprandial hyperglycemia, namely glycogen synthesis is reduced, and production of glucose is increased (Figure 2). Therefore, we proposed two approaches by which medicinal plants could ameliorated hyperglycemia through enhancing hepatic glucose metabolism: improving the function of insulin in the liver by inhibiting the enzyme PTP-1B and modulating the hepatic production/storage of glucose by regulating the enzymes involved in gluconeogenesis, glycogenolysis, and glycogenesis.

### Inhibition of Protein Tyrosine Phosphatase 1B

The modification of proteins through phosphorylation and dephosphorylation of tyrosine residues represents one of the main mechanisms of cell signaling regulation (Alonso et al., 2016), which is carried out by two superfamilies of enzymes: protein tyrosine kinases (PTKs), and PTPs. In this regard, the classical PTP subfamily possess a domain of 240–250 amino acids characterized by a conserved site that exhibits a catalytic mechanism based on cysteine (Denu and Dixon, 1998). Specifically, the enzyme PTP-1B is a classic intracellular PTP widely distributed in mammalian tissues that is anchored on the cytoplasmic side of the endoplasmic reticulum membrane. Despite its localization, the PTP-1B enzyme can access its substrates located on the surface of the plasma membrane during endocytosis, biosynthesis, and by the movement of the endoplasmic reticulum towards the plasma membrane in specific regions (Bakke and Haj, 2015).

Since its first isolation from the human placenta in 1988 by Tonks et al., 1988 PTP-1B has become an attractive research object due to its direct link with the etiopathogenesis of insulin resistance. In addition to the processes promoted by the obesogenic inflammatory environment, such as the serine/threonine phosphorylation of IR and IRS, and their proteasomal degradation (Mlinar et al., 2007; Ahmed et al., 2021), the dephosphorylation of these components by PTP-1B has also been implied to the termination of the insulin signal (Ahmad et al., 1995; Kenner et al., 1996; Chen et al., 1997).

Experimental data obtained from various studies have shown that the PTP-1B enzyme is one of the main negative regulators of the insulin signaling pathway. For instance, studies performed in PTP-1B knock-out mice have been shown that the absence of this enzyme produces healthy organisms that exhibit enhanced insulin sensitivity, protection against the weight gain generated by high-fat diet, and increased hepatic phosphorylation of IR and IRS after an intraperitoneal insulin injection (Elchebly et al., 1999; Klaman et al., 2000). On the other hand, it has been reported an increased PTP-1B activity in hepatic cytosolic fractions isolated from streptozotocin (STZ)-hyperglycemic rats (Meyerovitch et al., 1989), while augmented hepatic microsomal enzyme activity, content of protein, and mRNA levels have only been observed after 2 weeks of insulin treatment in these insulinopenic organisms, suggesting that elevated insulin levels are necessary to modify PTP-1B content and activity, namely hyperinsulinemia caused by insulin resistance may lead to altered PTP-1B expression and activity (Ahmad and Goldstein, 1995). Additionally, it has also been shown that insulin rises hepatic microsomal PTP-1B activity in rat hepatoma cells (Hashimoto and Goldstein, 1992). Likewise, abnormal expression and activity of PTP-1B have been reported in skeletal muscle of insulin-resistant obese people (Ahmad et al., 1997), as well as in non-obese Goto-Kakizaki rats with spontaneously generated insulin resistance (Dadke et al., 2000), and in STZ-hyperglycemic rats fed with high-fat diet (Wu et al., 2005).

Based on the aforementioned, the PTP-1B inhibition represents a good therapeutic target for the treatment of insulin resistance-related diseases, such as DM2 (Zhang et al., 2006). Hence, an arsenal of molecules with inhibitory capacity of PTP-1B activity has been generated in recent years. The methodological approaches that have been applied are the rational design of synthetic phospho-(tyrosine)-mimetic molecules to be used as competitive inhibitors, considering the structural characteristics of the protein, and the search for molecules from natural sources (Sun et al., 2018). The latter is based on the statement that nature has a great variety of structures that present diverse pharmacological effects (Atanasov et al., 2021), so natural products can be used as a starting point for the creation of powerful inhibitors.

Table 1 summarizes all medicinal plants and their identified compounds that have proved to inhibit the activity or expression of PTP-1B since 2015. It was obtained a total of 125 medicinal plants used in various traditional medicine systems around the world, mainly represented in eastern folk, such as Chinese and Vietnamese. *Morus alba* L. (Moraceae), a plant used in the traditional Chinese system, has been the most evaluated for this purpose. In addition to direct PTP-1B activity inhibition and molecular docking studies, some extracts and compounds were assessed to improve glucose and lipid metabolism *in vivo*, such as lowering blood glucose levels, improved insulin resistance and glucose intolerance, and improved lipid profile. Furthermore, their effect on glucose uptake and phosphorylation of some components of insulin signaling, such as IR, IRS, and Akt, was evaluated in cell cultures under insulin-resistant conditions.

**TABLE 1:** Medicinal plants and their phytochemicals with PTP-1B inhibitory capacity.

Medicinal plant (scientific name [Family]/Traditional medicine system or places where it is used)	Part/Extract	Isolated compounds	Experiment/Outcome	References
*Acmella paniculata* (Wall. ex DC.) R.K.Jansen [Asteraceae]/Indonesian	Aerial parts/EtOH	N-isobutyl-2E-decenamide	*In vitro*: PTP-1B enzyme assay/IC_50_ = 24 µM	Abdjul et al. (2018)
*Agrimonia pilosa* Ledeb. [Rosaceae]/Chinese	Aerial parts/EtOH	Apigenin-7-O-b-D-glucuronide-6″-methyl ester Quercetin-3-O-b-D-glycoside Kaempferol Kaempferol-3-O-a-l-rhamnoside b-sitosterol Ursolic acid Tormentic acid Methyl 2-hydroxyl tricosanoate Palmitic acid	*In vitro*: PTP-1B enzyme assay/IC_50_ = 14.35, 27.73, 42.93, 12.16, 49.78, 3.47, 0.5, 36.39, 0.1 µM	Na et al. (2016)
		Apigenin 7-O-b-D-glucuronide Ellagic acid Agritannin	*In vitro*: PTP-1B enzyme assay/IC_50_ = 7.14, 7.73, 17.03 µM	Nguyen et al. (2017)
*Akebia quinata* (Thunb. ex Houtt.) Decne. [Lardizabalaceae]/Chinese	Stems/MeOH	Cyrtophyllones B Uncinatone 3-*O*-α-l-arabinopyranosyl olean-12-en-28-oic acid 3-*O*-[β-d-glucopyranosyl (1–4)-α-l-arabinopyranosyl)]olean-12-en-28-oic acid 2α,3α,23-trihydroxyoleane-12-en-28-oic acid	*In vitro*: PTP-1B enzyme assay/IC_50_ = 6.77, 5.41, 4.08, 21.8, 7.78 µM	An et al. (2016)
*Allium cepa* L. [Amaryllidaceae]	Outer skins/MeOH	Cepadial B, C Cepabifla A–C Cepadial D	*In vitro*: PTP-1B enzyme assay/IC_50_ = 22.55, 22.33, 17.01, 24.07, 14.29, 1.68 µM	Vu et al. (2020)
*Allophylus cominia* (L.) Sw. [Sapindaceae]/Cuban	Leaves/MeOH	Pheophytin A, B	*In vitro*: PTP-1B enzyme assay/Activity inhibition by 65 and 57% at 30 μg/ml *In vitro*: cell culture (L6 myotubes)/↑insulin-dependent glucose uptake (pheophytin extract) *In vitro*: cell culture (3T3-L1)/↓lipid accumulation on the differentiation phase, ↓lipid droplets (phaeophytin extract)	Semaan et al., 2017, 2018
*Angelica decursiva* (Miq.) Franch. & Sav. [Apiaceae]/Korean	Whole plant/MeOH	*cis*-3′-Acetyl-4′-angeloylkhellactone Isorutarine	*In vitro*: PTP-1B enzyme assay/IC_50_ = 86.95, 80.09 µM	Yousof Ali et al. (2015)
*Anoectochilus chapaensis* Gagnep. [Orchidaceae]/Chinese	Whole plant/EtOH	Friedelin Sorghumol Epifriedelanol Friedelane 2a,3b-dihydroxyolean-12- en-23, 28, 30-trioic acid Quercetin Isorhamnetin Isorhamnetin-3-O-b-D-glucoside Isorhamnetin-3-O-b-d-rutinoside	*In vitro*: PTP-1B enzyme assay/IC_50_ = 6.21, 3.5, 3.75, 4.6, 2.65, 5.63, 1.75, 1.16, 1.2 µM	Cai et al. (2015)
*Artocarpus nanchuanensis* S.S.Chang S.C.Tan & Z.Y.Liu [Moraceae]/Chinese	Stems/EtOH	Hypargystilbene B, D, E	*In vitro*: PTP-1B enzyme assay/IC_50_ = 3.23, 37.31, 2.53 nM	Zhang et al. (2015)
*Artocarpus styracifolius* Pierre [Moraceae]/Chinese	Roots/EtOH	(±)-Styrastilbene A Styrastilbene B (±)-Styrastilbene C	*In vitro*: PTP-1B enzyme assay/IC_50_ = 4.52, 2.4, 8.23 µM	Li et al. (2019b)
*Astragalus mongholicus* Bunge [Fabaceae]/Chinese	Root/Aqueous	Astragaloside IV	*In vitro*: PTP-1B enzyme assay/IC_50_ = 10.34 µM *In vitro:* cell culture (insulin-resistant HepG2)/↑glucose consumption, ↓PTP-1B, ↑pIR, ↑pIRS1 protein levels	Zhou et al. (2021)
*Bidens pilosa L.* [Asteraceae]/Chinese	Whole plant/Aqueous In combination with *Euonymus alatus* (Thunb.) Siebold [Celastraceae] winged branchlet *Coptis chinensis* Franch. [Ranunculaceae] rhizome *Cornus officinalis* Siebold & Zucc. [Cornaceae] fruit *Ligustrum lucidum* W.T.Aiton [Oleaceae] fruit *Scrophularia ningpoensis* Hemsl. [Scrophulariaceae] root	Full extract	*In vivo:* hypertensive rats fed with HFD (2020 mg/kg b.w.)/prevention of increased body weight, ↓triglycerides, ↓LDL, ↓insulin resistance, ↑glucose tolerance, ↓ PTP-1B expression in adipose tissue	Zhu et al. (2018)
*Bistorta officinalis* Delarbre [Polygonaceae]/Chinese	Rhizome/EtOAc	Full extract	*In vitro*: PTP-1B enzyme assay/IC_50_ = 17.43 μg/ml	Zhao et al. (2019b)
*Boehmeria nivea* (L.) Gaudich. [Urticaceae]/Chinese	Root/EtOAc	Full extract Hederagenin Pomolic acid	*In vitro*: PTP-1B enzyme assay/IC_50_ = 20.19 μg/ml, 9.53, 4.89 µM	Zhao et al. (2019b)
*Camellia crapnelliana* Tutcher [Theaceae]/Chinese	Twigs and leaves/MeOH	Camellianol B, C, E–G A_1_- barrigenol 22-O-angeloyl-A_1_-barrigenol Camelliagenin A 16-O-acetylcamelliagenin A 3β,11α,13β-trihydroxyolean-12-one α-amyrin Lupeol 3β,20-dihydroxylupane	*In vitro*: PTP-1B enzyme assay/IC_50_ = 4.87, 7.4, 20.03, 14.36, 11.08, 16.79, 2.56, 8.93, 10.16, 1.34, 19.26, 3.68, 12.44 µM	Xiong et al. (2017)
*Cassia fistula* L. [Fabaceae]/Vietnamese	Leaves/EtOAc	Full extract	*In vitro*: PTP-1B enzyme assay/IC_50_ = 24.1 μg/ml	Trinh et al. (2017a)
*Catharanthus roseus* (L.) G.Don [Apocynaceae]/Malaysia, India, China, South Africa, and Mexico	Leaves/DCM	Vindogentianine	*In vitro*: PTP-1B enzyme assay/IC_50_ = 15.28 μg/ml *In vitro:* cell culture (β-TC6, C2C12)/↑glucose uptake	Tiong et al. (2015)
*Cedrus deodara* (Roxb. ex D.Don) G.Don [Pinaceae]/Aryuveda	Needles/Essential oil	Caryophyllene oxide	*In vitro*: PTP-1B enzyme assay/IC_50_ = 31.32 µM	Wang et al. (2017a)
*Centella asiatica* (L.) Urb. [Apiaceae]/Jamu	Aerial parts/Aqueous	Full extract	*In vitro*: PTP-1B enzyme assay/IC_50_ = 13.2 μg/ml	Saifudin et al. (2016b)
*Chaenomeles japonica* (Thunb.) Lindl. ex Spach [Rosaceae]/Japanese and Chinese	Fruits/acetone	Polyphenolic extract	*In vitro:* cell culture (HepG2)/↓PTP-1B mRNA expression level, ↓PEPCK mRNA expression level, ↑GLUT4 mRNA expression level, ↑IRS-2 mRNA expression level, ↑pAMPK, ↑glycogen synthesis, ↓glucose production	Zakłos-Szyda and Pawlik, (2018)
*Cinnamomum osmophloeum* Kaneh. [Lauraceae]/Taiwan	Twigs and leaves/acetone	Full extract *n*-hexane soluble fraction ethyl acetate soluble fraction *n*-butanol soluble fraction water soluble fraction	*In vitro*: PTP-1B enzyme assay/IC_50_ = 1.9, 3.2, 2, 1.7, 1.9 μg/ml	Lin et al. (2016)
*Cipadessa baccifera* (Roth) Miq. [Meliaceae]/Chinese	Leaves/EtOH	Cipacinoid A	*In vitro*: PTP-1B enzyme assay/IC_50_ = 16.7 µM	Yu et al. (2016a)
*Clausena sanki* (Perr.) Molino [Rutaceae]/Chinese	Fruits/EtOH	Clausenanisines A–C, E, F Euchrestifoline Dihydromupamine Clauraila B Kurryame Clausenaline F 3-formyl-1-hydroxycarbazole Clausine Z, I Clauszoline N, M	*In vitro*: PTP-1B enzyme assay/IC_50_ = 0.58, 0.87, 28.79, 27.96, 2.47, 1.28, 15.26, 23.89, 27.93, 28.42, 4.36, 5.39, 3.96, 24.43, 26.37 µM	Liu et al. (2021)
*Coptis chinensis* Franch. [Ranunculaceae]/Chinese	Rhizome/MeOH	Berberine Epiberberine Magnoflorine Coptisine	*In vitro*: PTP-1B enzyme assay/IC_50_ = 16.43, 21.19, 28.14, 51.04 µM	Choi et al. (2015)
*Coreopsis tinctoria* Nutt. [Asteraceae]/North American and Chinese	Capitula/EtOH	Butin Taxifolin 7,3′,4′-trihydroxyflavone Quercetagitin-7-O-b-D-glucoside	*In vitro*: PTP-1B enzyme assay/IC_50_ = 20.92, 7.73, 27.93, 24.5 µM	Begmatov et al. (2020)
*Cymbopogon nardus* (L.) Rendle [Poaceae]/Jamu	Leaves/Aqueous	Full extract	*In vitro*: PTP-1B enzyme assay/IC_50_ = 10.63 μg/ml	Saifudin et al. (2016b)
*Dioscorea bulbifera* L. [Dioscoreaceae]/Chinese	Rhizome/EtOAc	Full extract 9,10-Dihydro-2,4,6,7-phenanthrenetetrol [1,1'-Biphenanthren]-2,2',3,3',6,6',7,7'-octaol Cassigarol D	*In vitro*: PTP-1B enzyme assay/IC_50_ = 32.21 μg/ml, 23.79, 3.36, 13.16 µM	Zhao et al. (2019b)
*Dracaena cochinchinensis* (Lour.) S.C.Chen [Asparagaceae]/Chinese	Red resin/MeOH	Biflavocochin B, F, G	*In vitro*: PTP-1B enzyme assay/IC_50_ = inhibition of 75.8, 66.7, 74.9% at 10 µM	Lang et al. (2020)
*Duranta erecta* L. [Verbenaceae]/Aryuveda	Whole plant/EtOH	Full extract	*In silico:* Network pharmacology/phytoconstituents targeting PTP-1B *In silico:* molecular docking/PTP-1B binding energy: 8.9 kcal/mol (durantanin I) *In vivo:* diabetic rats (100, 200, and 400 mg/kg b.w.)/chronic hypoglycemic effect, ↓HbA1c, ↑glucose tolerance, ↓G6Pase and FBPase activity, ↑hexokinase activity, ↓triglycerides, LDL, VLDL, and total cholesterol, ↑HDL, ↑hepatic glycogen content, ↑glucose uptake in isolated rat hemidiaphragm	Khanal and Patil, (2020)
*Elaeocarpus grandiflorus* Sm. [Elaeocarpaceae]/Jamu	Fruits/Aqueous	Full extract	*In vitro*: PTP-1B enzyme assay/IC_50_ = 6.9 μg/ml	Saifudin et al. (2016b)
*Elephantopus scaber* L. [Asteraceae]/Jamu	Aerial parts/Aqueous	Full extract	*In vitro*: PTP-1B enzyme assay/IC_50_ = 2.64 μg/ml	Saifudin et al. (2016b)
*Eleutherococcus senticosus* (Rupr. & Maxim.) Maxim. [Araliaceae]/Chinese	Stems/MeOH	(7S,8R)-3-hy- droxyl-4-methoxyl-balanophonin (7S,8R)-5-methoxyl-balanophonin Balanophonin Curcasinlignan A–C	*In vitro*: PTP-1B enzyme assay/IC_50_ = 15.2, 12.6, 16.1, 17.1, 31, 29.4 µM	Li et al. (2017)
*Epimedium koreanum* Nakai [Berberidaceae]/Chinese	Aerial parts/MeOH	Icaritin Icariside II	*In vitro*: PTP-1B enzyme assay/IC_50_ = 11.59, 9.94 µM	Kim et al. (2017a)
*Eremophila bignoniiflora* (Benth.) F. Muell. [Scrophulariaceae]/Australian	Leaves/EtOAc	7-hydroxy-6-methyl-4-oxo-2-(3-(5-oxo-2,5- dihydrofuran-3-yl)propyl)hept-5-en-1-yl (E)-3-(3,4-dihydroxyphenyl) acrylate Galangin 3-methyl ether	*In vitro*: PTP-1B enzyme assay/IC_50_ = 52.4, 41.4 µM	Zhao et al. (2019a)
*Eremophila lucida* Chinnock [Scrophulariaceae]	Leaves/EtOAc	5-hydroxyviscida-3,14-dien-20-oic acid	*In vitro*: PTP-1B enzyme assay/IC_50_ = 42 µM	Tahtah et al. (2016)
*Eremophila oppositifolia* R.Br. [Scrophulariaceae]/Australian	Leaves/CH₃CN	Type B dimeric fatty acids related to the branched-chain fatty acid (2E,4Z,6E)-5-(acetoxymethyl)tetradeca-2,4,6-trienoic acid (Compounds 9, 12, 13a, 13b)	*In vitro*: PTP-1B enzyme assay/IC_50_ = 24, 2.4, 12, 12 µM	Pedersen et al. (2020)
*Eriobotrya japonica* (Thunb.) Lindl. [Rosaceae]/Chinese	Leaves/EtOH	Extract of triterpenoid acids (maslinic acid, corosolic acid, oleanolic acid, and ursolic acid)	*In vivo:* insulin-resistant mice (200 mg/kg b.w.)/↓insulin resistance, ↑glucose tolerance, ↓triglycerides, LDL, VLDL, and total cholesterol, ↑HDL; in liver: ↑PPARg, GLUT2, and glucokinase mRNA expression levels, ↓PTP-1B mRNA expression levels	Li et al. (2020b)
*Eucalyptus robusta* Sm. [Myrtaceae]/Chinese	Leaves/EtOH	Eucarobustol A–I Macrocarpal C	*In vitro*: PTP-1B enzyme assay/IC_50_ = 1.3, 4.3, 4.3, 2.9, 4.1, 5.6, 1.8, 3.0, 1.6, 4.5 µM	Yu et al. (2016b)
*Euphorbia hirta* L. [Euphorbiaceae]/Vietnamese	Whole plant/EtOAc and *n*-BuOH	Full extracts	*In vitro*: PTP-1B enzyme assay/IC_50_ = 29.2, 38.3 μg/ml	Trinh et al. (2017a)
*Ficus deltoidea* Jack [Moraceae]/Malay	Leaves/EtOH	70% EtOH extract Lupeol 3β,11β-dihydroxyolean-12-en-23-oic acid	*In vitro*: PTP-1B enzyme assay/IC_50_ = 92%, 2.88, 4.55 µM *In vivo:* diabetic rats (125, 250, and 500 mg/kg b.w. of 70% EtOH extract)/chronic hypoglycemic effect, ↓triglycerides, LDL, and total cholesterol, ↑HDL; in liver: ↑GLUT2 levels, ↓PEPCK, G6Pase, and PTP-1B mRNA expression levels	Abdel-Rahman et al. (2020)
*Ficus racemosa* L. [Moraceae]/Vietnamese	Fruit/EtOAc	Isoderrone Derrone Alpinumisoflavone Mucusisoflavone B	*In vitro*: PTP-1B enzyme assay/IC_50_ = 22.7, 12.6, 21.2, 2.5 µM	Trinh et al. (2017a)
*Garcinia mangostana* L. [Clusiaceae]/Southeast Asia and India	Fruits/EtOH	γ-Mangostin 8-Deoxyartanin 1,3,7-Trihydroxy-2,8-di-(3-methylbut-2-enyl)- xanthone α-Mangostin Garcinone E 9-Hydroxycalabaxanthone	*In vitro*: PTP-1B enzyme assay/IC_50_ = 0.86, 1.57, 3.28, 1.34, 0.43, 12.89 µM	Hu et al. (2021)
*Garcinia oblongifolia* Champ. ex Benth [Clusiaceae]/Vietnamese	Twigs/EtOAc	Norcowanin	*In vitro*: PTP-1B enzyme assay/IC_50_ = 14.1 µM	Trinh et al. (2017b)
*Geranium collinum* Stephan ex Willd. [Geraniaceae]/Chinese and Tajik	Root/EtOH In combination with *Hypericum scabrum* aerial parts (ratio: 7:3)	Full extract	*In vitro*: PTP-1B enzyme assay/IC_50_ = 0.48 μg/ml *In vitro:* cell culture (L6 myotubes, in the presence of insulin)/↓PTP-1B protein, ↑IR, ↑pAkt, ↑pIRS-1, ↑pGSK3β, ↑pAMPK, ↑glucose consumption	Edirs et al. (2018)
	Roots/EtOH	3,3′,4,4′-Tetra-O-methylellagic acid 3,3′-Di-O-methylellagic acid Caffeic acid Quercetin Catechin Epicatechin Corilagin	*In vitro*: PTP-1B enzyme assay/IC_50_ = 21.64, 6.26, 35.81, 2.19, 0.62, 0.23, 0.87 µM	Numonov et al. (2017)
*Glycyrrhiza inflata* Batalin [Fabaceae]/Japanese and Chinese	Roots and rhizomes/EtOAc	Licoagrochalcone A Kanzonol C Glyurallin B Gancaonin H 2′-hydroxyisolupalbigenin Gancaonin Q Glisoflavanone Glabrol Macarangaflavanone B	*In vitro*: PTP-1B enzyme assay/IC_50_ = 0.97, 0.45, 4.5, 1.48, 0.5, 0.55, 0.84, 0.31, 1.03 µM	Lin et al. (2017)
*Glycyrrhiza uralensis* Fisch. ex DC. [Fabaceae]/Chinese	Rhizomes/EtOH	Licochalcone A Licoflavone B	*In vitro*: PTP-1B enzyme assay/IC_50_ = 27.95, 15.62 µM	Guo et al. (2015)
		Isoangustone A Angustone A	*In vitro*: PTP-1B enzyme assay/IC_50_ = 3, 0.4 µM	Ji et al. (2016)
*Glyptostrobus pensilis* (Staunton ex D.Don) K.Koch [Cupressaceae]/Chinese	Trunk barks/MeOH	Spiropensilisol A, B 3-*epi*-larixinol 3,2′-*epi*-larixinol Abiesinol F Larixinol (Abiesinol E)	*In vitro*: PTP-1B enzyme assay/IC_50_ = 3.3, 11.2, 17.1, 4.6, 12.9, 8.1 µM	Xiong et al. (2020)
*Gymnema latifolium* Wall. ex Wight [Apocynaceae]/Vietnamese	Aerial parts/EtOH	Gymlatinoside GL2, GL3	*In vitro*: PTP-1B enzyme assay/IC_50_ = 22.66, 19.83 µM	Pham et al. (2020)
*Gynostemma pentaphyllum* (Thunb.) Makino [Cucurbitaceae]/Chinese	Aerial parts/EtOH	Gypenoside 2–6	*In vitro*: PTP-1B enzyme assay/IC_50_ = 18.2, 23.5, 28.6, 8.2, 12.5 µM	Wang et al. (2017b)
*Helicteres isora* L. [Malvaceae]/Jamu	Gum/Aqueous	Full extract	*In vitro*: PTP-1B enzyme assay/IC_50_ = 3.49 μg/ml	Saifudin et al. (2016b)
*Houttuynia cordata* Thunb. [Saururaceae]/Korea, Japan, India, and China	Aerial parts/EtOH	3-hydroxy-1,2-dimethoxy-5-methyl-5H-dibenzoindol-4- one 4-hydroxy-1,2,3-trimethoxy- 7H-dibenzo-quinolin-7-one 7-oxodehy- droasimilobine Cepharadione B	*In vitro*: PTP-1B enzyme assay/IC_50_ = 1.254, 2.016, 2.672, 1.862 µM	Ma et al. (2017)
*Hypericum longistylum* Oliv. [Hypericaceae]/Chinese	Aerial parts/MeOH	Longistylione A–D	*In vitro*: PTP-1B enzyme assay/IC_50_ = 18.87, 16.76, 24.56, 15.96 µM	Cao et al. (2017)
*Hypericum perforatum* L. [Hypericaceae]/Chinese	Aerial parts/EtOH	Full extract	*In vitro*: PTP-1B enzyme assay/IC_50_ = 1.08 μg/ml *In vivo:* insulin-resistant mice (50 and 200 mg/kg b.w.)/↓PTP-1B expression, ↑hepatic pAkt, ↑hepatic pIRS-1, ↑glucose tolerance, ↑insulin sensitivity, ↓triglycerides, improvement of lipid metabolism	Tian et al. (2015)
*Hypericum scabrum* L. [Hypericaceae]/Chinese	Aerial parts/EtOH	Quercetin	*In vitro*: PTP-1B enzyme assay/IC_50_ = 2.19 µM	Jiang et al. (2015)
*Iris sanguinea* Hornem. [Iridaceae]/Chinese	Seeds/MeOH	Kikkanol F monoacetate	*In vitro*: PTP-1B enzyme assay/IC_50_ = 7.3 µM *In vitro:* cell culture/↑glucose uptake (3T3-L1), ↑pAMPK (C2C12)	Yang et al. (2017)
*Juniperus chinensis* L. [Cupressaceae]/Chinese	Heartwood/MeOH	a-methyl artoflavanocoumarin	*In vitro*: PTP-1B enzyme assay/IC_50_ = 25.27 µM *In vitro:* cell culture (insulin-resistant HepG2)/↓PTP-1B protein, ↑pPI3K, ↑pAkt, ↑pERK1, ↑insulin-stimulated glucose uptake	Jung et al. (2017)
*Kandelia candel* (L.) Druce [Rhizophoraceae]/Vietnamese	Bark/EtOAc and *n*-BuOH	Full extracts	*In vitro*: PTP-1B enzyme assay/IC_50_ = 12.9, 0.02 μg/ml	Trinh et al. (2017a)
*Lagerstroemia speciosa* (L.) Pers. [Lythraceae]/Vietnamese	Leaves/*n*-BuOH	Full extract	*In vitro*: PTP-1B enzyme assay/IC_50_ = 19.6 μg/ml	Trinh et al. (2017a)
*Lantana camara* L. [Verbenaceae]/Indonesian and Japanese	Aerial parts/EtOH	24- hydroxy-lantadene B 3-hydroxy-lantadene C Icterogenin 4-epi- hederagonic acid Oleanolic acid 22b-oleanolic acid 3b-hydroxy-lantadene A 3b-hydroxy-lantadene B 22-hydroxy-oleanonic acid Lantadene B, A Oleanonic acid Lantadene D Pomonic acid Pomolic acid Lantanilic acid Camaric acid Lantanolic acid	*In vitro*: PTP-1B enzyme assay/IC_50_ = 7.3, 7.3, 11, 8.1, 2, 7.9, 7.2, 5.1, 6.9, 5.5, 5.2, 6.9, 7.9, 10.5, 10.6, 7.5, 5.1, 13 µM	Abdjul et al. (2017)
*Lanxangia tsao-ko* (Crevost & Lemarié) M.F. Newman & Škorničk [Zingiberaceae]/Asian countries	Fruits/EtOH	Tsaokoflavanol F, J, K, L, S	*In vitro*: PTP-1B enzyme assay/IC_50_ = 56.4, 75.1, 80.4, 73, 69.8 µM *In vivo: db/db* mice (200 and 400 mg/kg b.w.)/chronic hypoglycemic effect (full extract)	He et al. (2020)
*Leonurus sibiricus* L. [Lamiaceae]/Mongolian	Aerial parts/MeOH	Full extract	*In vitro*: PTP-1B enzyme assay/Activity inhibition by 40% at 10 μg/ml *In vitro:* cell culture (C2C12, in the presence of insulin)/↑glucose uptake	Pitschmann et al. (2016)
*Lithocarpus polystachyus* (Wall. ex A.DC.) Rehder [Fagaceae]/Chinese	Leaves/MeOH	Full extracts from five localities in China	*In vitro*: PTP-1B enzyme assay/IC_50_ = inhibition rate ranging from 84.3 to 90.3% at 1.25 mg/ml	Meng et al. (2020)
*Litsea cubeba* (Lour.) Pers. [Lauraceae]/Chinese	Twigs/EtOAc	(+)-9,9′-O-di-(E)-feruloyl-5,5′-dimethoxy secoisolariciresinol	*In vitro*: PTP-1B enzyme assay/IC_50_ = 13.5 µM	Li et al. (2019c)
*Lonicera japonica* Thunb. [Caprifoliaceae]/Chinese	Flower buds/EtOH	Lonjaponspiroside A, B	*In vitro*: PTP-1B enzyme assay/IC_50_ = 6.14, 8.42 µM	Liu et al. (2016)
*Ludwigia octovalvis* (Jacq.) P.H.Raven [Onagraceae]/Vietnamese	Aerial parts/EtOAc and *n*-BuOH	Full extracts	*In vitro*: PTP-1B enzyme assay/IC_50_ = 16.9, 3.3 μg/ml	Trinh et al. (2017a)
*Macaranga denticulata* (Blume) Müll.Arg. [Euphorbiaceae]/Chinese	Twings and leaves/EtOH	Macdentichalcone 1-(5,7-dihydroxy-2,2,6-trimethyl-2H-1-benzopyran-8-yl)-3-phenyl-2-propen-1-one	*In vitro*: PTP-1B enzyme assay/IC_50_ = 21, 22 µM	Lei et al. (2016)
*Macleaya cordata* (Willd.) R.Br. [Papaveraceae]/Chinese	Aerial parts/EtOH	Macleayine	*In silico*: molecular docking	Sai et al. (2016)
*Maclura tricuspidata* Carrière [Moraceae]/Korean, Japanese, and Chinese	Leaves/Aqueous	Full extract	*In vitro*: PTP-1B enzyme assay/IC_50_ = 65 μg/ml *In vitro*: PTP-1B enzyme assay (protein chip screening method)/↓PTP-1B activity *In vitro*: cell culture (3T3-L1)/↓lipid droplets, ↑pIRS-1, ↑pAkt *In vivo:* obese mice (20 and 100 mg/kg b.w.)/↑hepatic pIRS-1, ↑hepatic pAkt ↓tryglicerides, ↓glucose tolerance	Kim et al. (2016)
	Root barks/MeOH	Cudratricusxanthone N 1,6,7-trihydroxy-2-(1,1-dimethyl-2-propenyl)-3-methoxyxanthone Cudratricusxanthone L, A Cudraxanthone L Macluraxanthone B Cudracuspixanthone A Cudraxanthone D, M Cudraflavanone D Euchrestaflavanone C Cudraflavone C Kuwanon C	*In vitro*: PTP-1B enzyme assay/IC_50_ = 2, 3, 3, 4.3, 4.6, 3.8, 1.9, 2.8, 3.5, 5.7, 12.3, 9.4, 13.6 µM	Quang et al. (2015)
*Magnolia aromatica* (Dandy) V.S.Kumar [Magnoliaceae]/Chinese	Twigs and leaves/EtOH	(1R,6S,7S)-1-hydroxy- cadin-4,9-dien-8-onea	*In vitro*: PTP-1B enzyme assay/IC_50_ = 83.5 µM	Wang et al. (2016b)
*Magnolia officinalis* Rehder & E.H.Wilson [Magnoliaceae]/Chinese	Root barks/MeOH	Full extract	*In vitro*: PTP-1B enzyme assay/IC_50_ = 55.96 μg/ml *In vitro:* cell culture (3T3-L1 and C2C12, in the presence of insulin)/↑pIRb, ↑pERK, ↑GLUT4 translocation *In vivo:* db/db mice (0.5 g/kg b.w.)/chronic hypoglycemic effect	Sun et al. (2015)
*Magnolia officinalis* var. *biloba* Rehder & E.H.Wilson [Magnoliaceae]/Chinese	Barks/EtOH	Magterpenoid A, C	*In vitro*: PTP-1B enzyme assay/IC_50_ = 1.44, 0.81 µM	Li et al. (2018)
		Magmenthane E, H	*In vitro*: PTP-1B enzyme assay/IC_50_ = 4.38, 3.8 µM	Li et al. (2019a)
	Root and stem bark/EtOH	(±)-Mooligomers B, D, E	*In vitro*: PTP-1B enzyme assay/IC_50_ = 0.47, 2.1, 0.35, 1.22, 0.89, 0.14 µM	Li et al. (2020a)
*Melaleuca leucadendra* (L.) L. [Myrtaceae]/Jamu	Fruit/MeOH	Betulinic acid Ursolic acid	*In vitro*: PTP-1B enzyme assay/IC_50_ = 1.5, 2.3 µM	Saifudin et al. (2016a)
	Leaves/Aqueous	Full extract	*In vitro*: PTP-1B enzyme assay/IC_50_ = 2.05 μg/ml	Saifudin et al. (2016b)
*Melicope pteleifolia* (Champ. ex Benth.) T.G. Hartley [Rutaceae]/Chinese	Roots/CH_2_Cl_2_/CH_3_OH (1:1)	Melicoptelin B1/B2, D1/D2, E	*In vitro*: PTP-1B enzyme assay/IC_50_ = 34.4, 55.2, 66.6 µM	Xu et al. (2019b)
*Momordica charantia* L. [Cucurbitaceae]	Fruits/EtOH	25- O-methylkaraviagein D (19R,23E)-5b,19-epox y-19,25-dimethoxycucurbita-6,23-dien-3b-ol	*In vitro*: PTP-1B enzyme assay/IC_50_ = 51.8, 54.95 µM	Yue et al. (2017)
*Morus alba* L. [Moraceae]/Chinese	Leaves/Aqueous	Full extract (mulberry leaves polysaccharide)	*In vivo:* insulin-resistant rats (200 mg/kg b.w.)/↑glucose tolerance, ↓insulin resistance, ↑hepatic glycogen synthesis, ↓hepatic PTP-1B expression, ↑hepatic pIRS-2, ↑hepatic pAkt-2, ↑hepatic PI3K	Ren et al. (2015)
	Fruits/EtOH	Full extract	*In vitro*: PTP-1B enzyme assay/IC_50_ = 11.89 μg/ml	Xiao et al. (2017b)
	Leave cell culture/EtOH	Morusalone A–D	*In vitro*: PTP-1B enzyme assay/IC_50_ = 2.51, 1.14, 0.35, 1.99 µM *In vitro:* cell culture (HepG2)/↑pIRβ and pAkt protein levels	Su et al. (2019)
		Morusalisin A–F	*In vitro*: PTP-1B enzyme assay/IC_50_ = 1.55, 2.24, 1.58, 1.52, 1.60, 1.14 µM	Su et al. (2020)
	Leaves/EtOH	mortatarin E 3′-geranyl-3-prenyl-2′,4′,5,7- tetrahydroxyflavone	*In vitro*: PTP-1B enzyme assay/IC_50_ = 4.53, 10.53 µM *In vitro:* cell culture (insulin-resistant HepG2)/↑glucose uptake, ↑glycogen synthesis, ↓PTP-1B, ↑IRS1, ↑IRS2, ↓GSK3β, and ↑GLUT4 mRNA expression levels, ↓PTP-1B, ↑pIRS1, ↑PI3K, ↑pAkt, and ↑GLUT4 protein levels (mortatarin E)	Niu et al. (2020)
	Root bark/MeOH	Morusalfuran A–C, F Morusalnol B Morusibene A	*In vitro*: PTP-1B enzyme assay/IC_50_ = 11.02, 8.92, 7.26, 18.02, 26.56, 17.64 µM	Ha et al. (2020)
	Roots/EtOAc	Kuwanon L Mulberrofuran G Moracenin B (Kuwanon G) Morusinol Sanggenon G Kuwanon C Moracenin A (Kuwanon H) Kuwanon T, F, M Morusin Mulberrofuran B Cyclomorusin Sanggenofuran A	*In vitro*: PTP-1B enzyme assay/IC_50_ = 21.67, 20.03, 13.07, 30.49, 10.87, 17.48, 4.04, 9.83, 9.52, 10.71, 19.63, 4.69, 13.28, 12.72 μg/ml	Zhao et al. (2018)
*Morus macroura* Miq. [Moraceae]/Chinese	Twigs/EtOH	Notabilisin E Taxifolin Hultenin	*In vitro*: PTP-1B enzyme assay/IC_50_ = 0.87, 5.3, 1.04 µM	Wang et al. (2015)
*Myrtus communis* L. [Myrtaceae]/Italian	Leaves/chloroform	3β-cis-*p*-coumaroyloxy-2α,23-dihydroxyolean-12-en-28-oic acid 3β-trans-*p*-coumaroyloxy-2α,24-dihydroxy-urs-12-en-28-oic acid Maslinic acid Corosolic acid Isomyrtucommulone B 3β-O-cis-*p*-coumaroyl-2α-hydroxy-urs-12-en-28-oic acid Jacoumaric acid Betulinic acid Oleanolic acid Ursolic acid	*In vitro*: PTP-1B enzyme assay/IC_50_ = 15.38, 14.89, 25.73, 12.21, 8.93, 26.67, 11.93, 16.05, 8.92, 14.93 µM	Liang et al. (2020)
*Nepenthes mirabilis* (Lour.) Druce [Nepenthaceae]/Vietnamese	Whole plant/EtOAc and *n*-BuOH	Full extracts	*In vitro*: PTP-1B enzyme assay/IC_50_ = 1.4, 0.4 μg/ml	Trinh et al. (2017a)
*Nigella sativa* L. [Ranunculaceae]/From Turkey to India	Aerial parts/MeOH	3-O-[α-l-Rhamnopyranosyl-(1→2)-α-l-arabinopyranpsyl]hederagenin	*In vitro*: PTP-1B enzyme assay/IC_50_ = 91.3 µM	Parveen et al. (2020)
*Nigella sativa* var. *hispidula* Boiss. [Ranunculaceae]/Uighur	Seeds/Petroleum ether	Nigelladine A–C	*In vitro:* cell culture (L6 myotubes, in the presence of insulin)/↓PTP-1B protein, ↑pAkt, ↑pIRS-1, ↑pGSK-3b, ↑pAMPK, ↑glucose consumption, ↑lactic acid production, ↑glycogen synthesis, ↑hexokinase activity	Tang et al. (2017)
*Orthosiphon aristatus* (Blume) Miq. [Lamiaceae]/Vietnamese and Indonesian	Aerial parts/MeOH	Siphonol B, D Orthosiphol B, F, G, I, N	*In vitro*: PTP-1B enzyme assay/IC_50_ = 8.18, 24.75, 9.84, 27.56, 3.82, 0.33, 1.6 µM *In vitro:* cell culture (3T3-L1)/↑glucose uptake	Nguyen et al. (2019)
*Ouret lanata* (L.) Kuntze [Amaranthaceae]/Ayurveda	Leaves/EtOH	Full extract	*In vitro*: PTP-1B enzyme assay/IC_50_ = 94.66 μg/ml *In vitro:* cell culture (L6 myotubes)/↑adipogenesis, ↑insulin-mediated glucose uptake *In vivo:* diabetic rats (500 mg/kg b.w.)/antihyperglycemic effect in OSTTs by 18.44%	Riya et al. (2015)
*Paeonia lactiflora* Pall. [Paeoniaceae]/Chinese	Seeds/EtOH	Paeonilactiflorol *trans*-gnetin H	*In vitro*: PTP-1B enzyme assay/IC_50_ = 27.23, 27.81 µM	Zhang et al. (2019a)
*Panax ginseng* C.A.Mey. [Araliaceae]/Eastern Asia	Stems, flowers, and fruits/EtOH	20(R)-25-methoxydammarane-3β,12β, 20-tetrol 20(R)-dammarane-3β,6α,12β, 20, 25-pentol 20(R)- protopanaxatriol 20(S)-panaxatriol 20(R)-protopanaxadiol	*In vitro*: PTP-1B enzyme assay/IC_50_ = 16.54, 10.07, 17.98, 21.02, 21.27 µM	Yang et al. (2016)
*Panax quinquefolius* L. [Araliaceae]/Chinese	Crude saponins/EtOH	20(S)-panaxadiol (20S,24R)-dammarane-20,24-epoxy-3 β,6 α, 12 β,25- tetraol 20(R)-dammarane-3 β, 12 β,20,25-tetraol 20(S)-dammarane-3 β,6 α, 12 β,20,25-pentol 20(R)-dammarane-3 β, 12 β,20,25-tetrahydroxy-3 β-O- β- d–glucopyranoside Oleanolic acid 20(S)-protopanaxadiol	*In vitro*: PTP-1B enzyme assay/IC_50_ = 27.23, 23.63, 10.39, 6.21, 5.91, 18.99, 13.38 µM	Han et al. (2020)
*Pandanus odorifer* (Forssk.) Kuntze [Pandanaceae]/Vietnamese	Fruit/EtOAc and *n*-BuOH	Full extracts	*In vitro*: PTP-1B enzyme assay/IC_50_ = 20.8, 40.4 μg/ml	Trinh et al. (2017a)
*Phyllanthus amarus* Schumach. & Thonn. [Phyllanthaceae]/Vietnamese	Whole plant/EtOAc	Full extract	*In vitro*: PTP-1B enzyme assay/IC_50_ = 74.4 μg/ml	Trinh et al. (2017a)
*Phyllanthus niruri* L. [Phyllanthaceae]/Jamu	Aerial parts/Aqueous	Full extract	*In vitro*: PTP-1B enzyme assay/IC_50_ = 10.99 μg/ml	Saifudin et al. (2016b)
*Phyllanthus* urinaria L. [Phyllanthaceae]/Vietnamese	Whole plant/EtOAc and *n*-BuOH	Full extracts	*In vitro*: PTP-1B enzyme assay/IC_50_ = 14, 10.8 μg/ml	Trinh et al. (2017a)
*Pithecellobium dulce* (Roxb.) Benth. [Fabaceae]/Vietnamese	Stem/EtOAc	Full extract	*In vitro*: PTP-1B enzyme assay/IC_50_ = 26.1 μg/ml	Trinh et al. (2017a)
*Prunus amygdalus* Batsch [Rosaceae]	Fruits/EtOH	Hexane fraction Chloroform fraction	*In vitro*: PTP-1B enzyme assay/IC_50_ = 9.66, 37.95 μg/ml	Qureshi et al. (2019)
*Psidium guajava* L. [Myrtaceae]/Worldwide	Leaves/EtOAc	Psiguadiol A–J	*In vitro*: PTP-1B enzyme assay/IC_50_ = 4.7, 11, 11.9, 10.7, 19.1, 18.9, 6.2, 9.2, 22.8, 22.8 µM	Hou et al. (2019)
	Leaves/EtOH	Jejuguajavone A, C	*In vitro*: PTP-1B enzyme assay/IC_50_ = 10.52, 9.4 µM	Ryu et al. (2021)
*Psydrax subcordatus* (DC.) Bridson [Rubiaceae]/African	Leaves and bark/EtOH	Subcordatanol I, III, IV	*In vitro*: PTP-1B enzyme assay/IC_50_ = 22.2, 8.9, 9.8 µM	Zhou et al. (2019)
*Pueraria montana* var. *lobata* (Willd.) Maesen & S.M.Almeida ex Sanjappa & Predeep [Fabaceae]/Chinese	Root/MeOH	Puerarin	*In silico:* molecular docking/PTP-1B binding energy: 6.4 kcal/mol	Ojo, (2021)
	Roots/EtOH	Lupeol Lupenone	*In vitro*: PTP-1B enzyme assay/IC_50_ = 38.89, 15.11 µM	Seong et al. (2016)
		Full extract	*In vitro*: PTP-1B enzyme assay/IC_50_ = 0.046 mg/ml *In vitro:* cell culture (insulin resistant HepG2 cells)/↑glucose uptake *In vivo:* insulin resistant mice (0.25, 0.5, 1, and 2 g/kg b.w.)/↓AUC in OGTTs by 5.7, 10, 20, and 21%	Sun et al. (2019)
*Quercus infectoria* G.Olivier [Fagaceae]/Jamu	Fruits/Aqueous	Full extract	*In vitro*: PTP-1B enzyme assay/IC_50_ = 4.68 μg/ml	Saifudin et al. (2016b)
*Quercus wutaishanica* Mayr [Fagaceae]/Chinese and Mexican	Acorn/EtOH	3-O-galloyloleanolic acid 23- acetoxy-3-O-galloyloleanolic acid 3-acetoxy-23-O-galloyloleanolic acid Oleanolic acid 3-O-galloylursolic acid Ursolic acid	*In vitro*: PTP-1B enzyme assay/IC_50_ = 2.10, 4.17, 4.52, 17.25, 1.86, 17.37 µM	Xu et al. (2018a)
	Leaves/EtOH	Quercetin-3-O-(2″-O-galloyl)-β-galactopyranoside Kaempferol-3-O-rutinoside Quercetin-3-O-rutinoside Quercetin Kaempferol Myricetin Dihydromyricetin 4′,5,7-trihydroxyflavanone 4′-methoxy-5′,5,7-trihydroxyflavanone Ellagic acid	*In vitro*: PTP-1B enzyme assay/IC_50_ = 5.56, 24.89, 20.56, 4.16, 3.92, 3.53, 9.58, 15.38, 20.16, 1.03 µM *In vitro:* cell culture (MIN6)/Protective effect on pancreatic b cells damaged by H_2_O_2_ (Quercetin-3-O-(2″-O-galloyl)-β-galactopyranoside)	Xu et al. (2018b)
*Reynoutria japonica* Houtt. [Polygonaceae]/Japanese, South Korean, and Chinese	Roots/EtOAc	(*trans*)-emodin-physcion bianthrone (*cis*)-emodin-physcion bianthrone	*In vitro*: PTP-1B enzyme assay/IC_50_ = 2.77, 7.29 µM	Zhao et al. (2017)
*Reynoutria multiflora* (Thunb.) Moldenke [Polygonaceae]/Chinese	Roots/EtOH	Multiflorumiside H–K	*In vitro*: PTP-1B enzyme assay/IC_50_ = 1.2, 1.7, 1.5, 4.6 µM	Yang et al. (2020)
*Rhizophora apiculata* Blume [Rhizophoraceae]/India	Leaves/EtOH	Glycosin	*In silico:* molecular docking/PTP-1B binding energy: 6.35 kcal/mol *In vivo:* diabetic rats (50 mg/kg b.w.)/↓blood glucose reduction by 25% in OGTTs, ↓chronic hypoglycemic effect, ↓HbA1c, ↓triglycerides, ↓cholesterol, ↑HDL, ↑hexokinase activity, ↓G6Pase activity, ↑FBPase activity	Selvaraj et al. (2016)
*Rhizophora mucronata* Poir. [Rhizophoraceae]/Vietnamese	Bark/EtOAc and *n*-BuOH	Full extracts	*In vitro*: PTP-1B enzyme assay/IC_50_ = 17.2, 1.8 μg/ml	Trinh et al. (2017a)
*Rhodiola rosea* L. [Crassulaceae]/Chinese	Whole plant/MeOH	Arbutin	*In vitro*: PTP-1B enzyme assay/IC_50_ = 20.5 µM	Yuan et al. (2021)
*Rhododendron fastigiatum* Franch. [Ericaceae]	Aerial parts/EtOH	(+)-fastinoid B (-)-fastinoid B Rubiginosin A (-)-rubiginosin A Grifolinone A	*In vitro*: PTP-1B enzyme assay/IC_50_ = 47, 54.9, 40.9, 49.2, 13 µM	Huang et al. (2019)
*Ricinus communis* L. [Euphorbiaceae]/Chinese	Rhizomes/EtOH	3α,19-dihydroxyl-ent-pimara-8 (14),15-diene	*In vitro*: PTP-1B enzyme assay/IC_50_ = 49.49% at 20 μg/ml	Zhang et al. (2019b)
*Rubus chingii* Hu [Rosaceae]/Chinese	Fruits/MeOH	Ursolic acid 2-oxopomolic acid 2α,19α-dihydroxy-3-oxo-urs- 12-en-28-oic acid	*In vitro*: PTP-1B enzyme assay/IC_50_ = 7.1, 23.7, 52.3 µM	Zhang et al. (2019c)
*Rubus idaeus* L. [Rosaceae]	Leaves/MeOH	Full extract	*In vitro*: PTP-1B enzyme assay/IC_50_ = 3.41 μg/ml	Li et al. (2016)
*Rubus occidentalis* L. [Rosaceae]	Fruits/EtOH	Cyanidin-3-O-xylosilutinoside Cyanidin-3-O -rutinoside Quercetin-3-O-rutinoside Ellagic acid	*In vitro*: PTP-1B enzyme assay/IC_50_ = 2.58, 1.88, 2.12, 0.03 µM	Xiao et al. (2017a)
*Salvia circinnata* Cav. [Lamiaceae]/Mexican	Aerial parts/Aqueous	6-hydroxyluteolin Pedalitin	*In vitro*: PTP-1B enzyme assay/IC_50_ = 80.1, 62 µM	Salinas-Arellano et al. (2020)
*Salvia miltiorrhiza* Bunge [Lamiaceae]/Chinese	Roots/EtOH	Cryptotanshinone Tanshinol B Tanshinonal 15,16-dihydrotanshinone I Tanshinone I Dehydrodanshenol A	*In vitro*: PTP-1B enzyme assay/IC_50_ = 5.5, 4.7, 37.6, 18.6, 27.1, 8.5 µM	Kim et al. (2017b)
	Selected compounds of Tangzhiqing herbal formula In combination with *Morus alba* L. (Moraceae) *Nelumbo nucifera* Gaertn. (Nelumbonaceae) *Crataegus pinnatifida* Bunge (Rosaceae) *Paeonia lactiflora* Pall. (Paeoniaceae)	Nuciferine Rutin 1-Deoxynojirimycin Salvianolic acid A Salvianolic acid C Danshensu Rosmarinic acid Tanshinone IIA Cryptotanshinone Dihydrotanshinone I Quercitrin Paeoniflori	*In silico:* molecular docking/PTP-1B binding energy: 26.49, 86.52, 17.57, 72.09, 95.07, 49.37, 54.44, 25.66, 33.08, 22.27, 83.52, 44.46 kcal/mol	Hao et al. (2020)
*Selaginella rolandi-principis* Alston [Selaginellaceae]/Vietnamese	Aerial parts/EtOH	Selaginolide A 2′-hydroxygenistein 6,7-dimethoxy-2′,4′-dihydroxyisoflavone	*In vitro*: PTP-1B enzyme assay/IC_50_ = 7.4, 23.02, 11.08 µM *In vitro:* cell culture (3T3-L1)/↑glucose uptake, ↑pIRS-1, ↑pPI3K, pAkt	Nguyen et al. (2021)
*Selaginella tamariscina* (P.Beauv.) Spring [Selaginellaceae]/Chinese	Aerial parts/MeOH	Selariscinin D Selariscinin E Amentoflavone Robustaflavone Cupressuflavone Taiwaniaflavone 3,8″-biapigenin	*In vitro*: PTP-1B enzyme assay/IC_50_ = 13.2, 9.8, 7.4, 6.2, 9.6, 5.4, 4.5 µM *In vitro:* cell culture (3T3-L1)/↑glucose uptake	Nguyen et al. (2015)
		Sellaginellin U–W Sellaginellin Selariscinin A	*In vitro*: PTP-1B enzyme assay/IC_50_ = 13.8, 14.5, 14.6, 15.9, 4.8 µM	Le et al. (2017)
*Selaginella uncinata* (Desv.) Spring [Selaginellaceae]/Chinese	Whole plant/EtOH	Uncinatabiflavone C 7-methyl ether Robustaflavone 4′-methyl ether Robustaflavone 7- methyl ether (2R) 2, 3-dihy- droamentoflavone Amentoflavone Bilobetin (2″S) chrysocauloflavone I Delicaflavone (2S) 2,3-dihydro- 5,5″,7,7″,4′-pentahydroxy-6,6″-dimethyl-[3′-O-4‴]-biflavone	*In vitro*: PTP-1B enzyme assay/IC_50_ = 7.7, 9.2, 9.8, 16.1, 10.6, 14.6, 5.5, 6.2, 4.6 µM *In vitro:* cell culture (insulin-resistant HepG2)/↑glucose uptake, ↑pIRS-1, ↑pPI3K, pAkt (Uncinatabiflavone C 7-methyl ether)	Xu et al. (2019a)
*Senna obtusifolia* (L.) H.S.Irwin & Barneby [Fabaceae]/Chinese	Seeds/MeOH	Physcion Chrysophanol Emodin Alaternin Obtusin Questin Chryso-obtusin Aurantio-obtusin 2-Hydroxyemodin-1 methylether	*In vitro*: PTP-1B enzyme assay/IC_50_ = 7.28, 5.86, 3.51, 1.22, 6.44, 5.69, 14.88, 27.19, 5.22 µM *In vitro:* cell culture (insulin-resistant HepG2)/↑insulin-stimulated glucose uptake (alaternin and emodin)	Jung et al. (2016)
*Silybum marianum* (L.) Gaertn. [Asteraceae]	Seeds/EtOAc	Taxifolin Dihydrokaempferol Dihydroquercetin-4′-methylether Kaempferol	*In vitro*: PTP-1B enzyme assay/IC_50_ = 24.23, 27.83, 21.30, 6.79 µM	Qin et al. (2017)
*Smilax china* L. [Smilacaceae]/Thai	Leaves/EtOH	Morin Kaempferol 7-O-α-l-rhamnoside Quercetin-4′-O-β-D-glucoside 4′-methoxy-5,7-dihydroxyflavone-(3-O-7″)-4’’’,5″,7″-trihydroxyflavone Partensein 1,3,6-trihydroxyxanthone	*In vitro*: PTP-1B enzyme assay/IC_50_ = 7.62, 10.80, 0.92, 2.68, 9.77, 24.17 µM	Zhao et al. (2016)
*Sophora flavescens* Aiton [Fabaceae]	Roots/EtOH	Sophobiflavonoid A, C	*In vitro*: PTP-1B enzyme assay/IC_50_ = 0.33, 0.35 µM	Yan et al. (2019)
*Symplocos cochinchinensis* (Lour.) S. Moore. [Symplocaceae]/Ayurveda	Bark/EtOH	Full extract	*In vivo:* insulin-resistant rats (250 and 500 mg/kg b.w.)/↓hepatic PTP-1B activity and expression, ↑hepatic pAkt, ↑hepatic pIRS-1, ↑glucose tolerance, ↑insulin sensitivity, ↓hepatic triglycerides, ↓expression of gluconeogenic enzymes	Antu et al. (2016)
*Syzygium cumini* (L.) Skeels [Myrtaceae]/Vietnamese, Ayurveda, Unani, and Chinese	Seeds/MeOH	Valoneic acid dilactone Rubuphenol Ellagic acid	*In vitro*: PTP-1B enzyme assay/IC_50_ = 9.37, 28.14, 25.96 µM	Sawant et al. (2015)
	Fruit/EtOAc	Full extract	*In vitro*: PTP-1B enzyme assay/IC_50_ = 27.5 μg/ml	Trinh et al. (2017a)
*Tetradium ruticarpum* (A.Juss.) T.G.Hartley [Rutaceae]/East Asia	Buds/MeOH	Schinifoline Intergrifoliodiol	*In vitro*: PTP-1B enzyme assay/IC_50_ = 24.3, 47.7 µM	To et al. (2021)
*Thonningia sanguinea* Vahl [Balanophoraceae]/Angola	Rhizomes/MeOH	2′-O- (3-O-galloyl-4,6-O-Sa-hexahydroxydiphenoyl-β-d-glucopyranosyl)-3-hydroxyphloretin 4′-O-(4,6-O-Sa-hexahydroxydiphenoyl-β-d-glucopyranosyl)- phloretin 2′-O-(3-O-galloyl-4,6-O-Sa- hexahydroxydiphenoyl-β-d-glucopyranosyl)phloretin Thonningianin B, A	*In vitro*: PTP-1B enzyme assay/IC_50_ = 24.7, 23.8, 19.3, 21.7, 4.4 µM *In vitro:* cell culture (HepG2)/↑insulin-stimulated IR phosphorylation (Thonningianin A)	Pompermaier et al. (2018)
*Tinospora sagittata* (Oliv.) Gagnep. [Menispermaceae]/Chinese	Rhizome/EtOAc	Full extract	*In vitro*: PTP-1B enzyme assay/IC_50_ = 38.5 μg/ml	Zhao et al. (2019b)
*Tradescantia spathacea* Sw. [Commelinaceae]/Vietnamese	Aerial parts/MeOH	Bracteanolide A Latifolicinin C, A Oresbiusin A	*In vitro*: PTP-1B enzyme assay/IC_50_ = 7.82, 6.80, 4.55, 6.38 µM	Vo et al. (2015)
*Ugni molinae* Turcz. [Myrtaceae]/Chilean	Leaves/EtOAc	Full extract Madecassic acid Myricetin	*In vitro*: PTP-1B enzyme assay/IC_50_ = 97.2% at 2 μg/ml (full extract) *In vivo:* Insulin resistant mice (20, 20, and 20 mg/kg b.w.)/↓PTP-1B mRNA expression in aorta (full extract), ↑glucose tolerance (full extract and madecassic acid), ↑aortic insulin sensitivity	Arancibia-Radich et al. (2019)
*Vaccinium myrtillus* L. [Ericaceae]	Fruits/EtOH	Full extract	*In vitro*: PTP-1B enzyme assay/IC_50_ = 6.96 μg/ml	Xiao et al. (2017b)
*Vaccinium uliginosum* L. [Ericaceae]	Fruits/EtOH	Full extract	*In vitro*: PTP-1B enzyme assay/IC_50_ = 3.06 μg/ml	Xiao et al. (2017b)
		Phenolic compounds Cyanidin-3-arabinoside Delphinidin-3-glucoside Cyanidin-3-galactoside Cyanidin-3-glucoside Malvidin-3-galactoside Petunidin-3-glucoside	*In vitro*: PTP-1B enzyme assay/IC_50_ = 8.91, 17.8, 19.8, 25.9, 34, 31.1 µM *In vitro:* cell culture (PTP-1B-overexpressed HepG2)/↑glucose consumption, ↑glycogen synthesis, ↓PTP-1B mRNA expression and protein level, ↑IRS1 and ↓GSK3β mRNA expression, ↑pIRS1, PI3K, pAkt, pAMPK, and pGSK3β protein level (cyanidin-3-arabinoside)	Tian et al. (2019)
		Procyanidin B1, B2	*In vitro*: PTP-1B enzyme assay/IC_50_ = 0.6, 4.79 µM *In vitro:* cell culture (HepG2)/↓PTP-1B mRNA expression and protein level	Li et al. (2021)
*Viburnum macrocephalum* Fortune [Viburnaceae]/Chinese	Fruits/EtOH	Viburmacrosides C Viburmacrosideand D (+)-8′-hydroxypinoresinol 4-O-b-D-glucoside (-)-olivil 4′-O-b-D-glucoside	*In vitro*: PTP-1B enzyme assay/IC_50_ = 25.8, 8.9, 28.7, 27.5 µM	Zhao et al. (2020)
*Vigna radiata* (L.) R.Wilczek [Fabaceae]	Seeds/Aqueous	Full extract	*In vitro*: PTP-1B enzyme assay/IC_50_ = 10 μg/ml *In vitro:* cell culture (insulin-resistant HepG2)/↑glucose uptake, ↓PEPCK and GSK3β mRNA expression level	Saeting et al. (2021)

EtOH: ethanolic extract; MeOH: methanolic extract; EtOAc; ethyl acetate extract; PTP-1B: protein tyrosine phosphatase 1B; OSTT: oral sucrose tolerance test; OGTT: oral glucose tolerance test; IR: insulin receptor; IRS1: insulin receptor substrate 1; IRS2: insulin receptor substrate 2; GSK3β: glycogen synthase kinase 3 beta; Akt: protein kinase B; PI3K: phosphoinositide 3-kinase; ERK1: extracellular signal-regulated kinase 1; HFD: high-fat diet; HDL: high-density lipoprotein; LDL: low-density lipoprotein; VLDL: very low-density lipoprotein; PEPCK: phosphoenolpyruvate carboxykinase; FBPase: fructose 1,6-bisphosphatase; G6Pase: glucose 6-phosphatase; GLUT2: glucose transporter 2; GLUT4: glucose transporter 4; AMPK: AMP-activated protein kinase; PPARγ: peroxisome proliferator-activated receptor gamma; HbA1c: glycated hemoglobin.

### Inhibition of Hepatic Glucose Output by Modulating Glucose Metabolism in Liver

The liver is a key organ that plays a crucial role in the regulation of blood glucose because it manages both storage and synthesis of glucose. The latter involves two metabolic pathways: glycogenolysis and gluconeogenesis, which constitute total hepatic glucose production (HGP) (Lee et al., 2015). Glycogenolysis consists of glycogen breakdown into glucose, being half of the basal HGP in fasting and decreasing the glycogen concentration at an almost linear rate during the first 22 h (Rothman et al., 1991; Cersosimo et al., 2018). In fasting, it is controlled by glucagon and epinephrine that activate glycogen phosphorylase (GP), the major enzyme responsible for digesting glycogen by releasing glucose 1-phosphate. In feeding condition, insulin inhibits glycogen breakdown and promotes glycogen synthesis through the activation of Akt and protein phosphatase 1 (PP1), leading the deactivation of both GP and glycogen synthase kinase-3 (GSK3), which in its active form (dephosphorylated), inactivates glycogen synthase (GS) (Han et al., 2016).

Gluconeogenesis, on the other hand, is defined as the production of glucose from a molecule that is not a carbohydrate. Its main substrates are pyruvate, glycerol, and amino acids such as alanine (Hanson and Owen, 2013). Another way to denote gluconeogenesis is as “reverse glycolysis” since both share not only substrates and final products, but also many enzymes. However, the direction of the reactions catalyzed in gluconeogenesis goes in the opposite direction, so the steps that are not shared with glycolysis can be determined as regulatory steps. These reactions are catalyzed by four rate-limiting enzymes: pyruvate carboxylase (PC), which is responsible for converting pyruvate into oxaloacetate; phosphoenolpyruvate carboxykinase (PEPCK), that converts oxaloacetate to phosphoenolpyruvate; fructose 1,6-bisphosphatase (FBPase), that dephosphorylates fructose 1,6-bisphosphate obtaining fructose 6-phosphate; and glucose 6-phosphatase (G6Pase), which is responsible for removing the phosphate group from glucose 6-phosphate, yielding *novo* synthesized glucose (Postic et al., 2004).

In the diabetic state, increased rates of HGP are observed as a result of an imbalance of various factors, such as the augmented availability of gluconeogenic substrates, the resistance of the liver to the action of insulin, and elevated levels of glucagon that activate HGP (Sharabi et al., 2015). Due to all these factors, the inhibition of HGP turns out to be an important therapeutic target for the reduction of hyperglycemia observed in T2D patients. In this regard, Table 2 summarizes the works made between 2015 and 2021 with extracts or natural products from 47 medicinal plants that showed to modulate hepatic glucose metabolism by inhibiting glucose production or promoting glycogen synthesis. As it can be observed, decreasing the expression of PEPCK and G6Pase is the principal mechanism related to gluconeogenesis inhibition, while phosphorylation of GSK3, promotion of GS activity, and inhibition of GP are the main mechanisms involved in glycogen breakdown and synthesis. Furthermore, although PI3K/Akt pathway stands out as a good pharmacological target to reduce insulin resistance, medicinal plants and their phytochemicals can also decrease HGP through AMP-activated protein kinase (AMPK).

**TABLE 2:** Medicinal plants and their phytochemicals capable to modulate hepatic glucose metabolism.

Medicinal plant (scientific name [Family]/Traditional medicine system or places where it is used)	Part/Extract	Isolated compounds	Experiment/Outcome	References
*Abelmoschus esculentus* (L.) Moench [Malvaceae]/Chinese	Whole plant/EtOH	Polysaccharides	*In vivo:* insulin-resistant mice (200 and 400 mg/kg b.w.)/↑pAkt and pGSK3β	Liao et al. (2019)
*Ageratina petiolaris* (Moc. & Sessé ex DC.) R.M.King & H.Rob. [Asteraceae]/Mexican	Aerial parts/Aqueous	Full extract	*In vivo:* diabetic rats (160 mg/kg b.w.)/↓Glucose production in PTTs *In vitro:* G6Pase inhibition assay/IC_50_ = 223 μg/ml	Mata-Torres et al. (2020)
*Aloe vera* (L.) Burm.f. [Asphodelaceae]/Aryuveda	Gel/EtOH	Carbohydrate fraction	*In vivo:* diabetic rats (27 and 54 mg/kg b.w.)/↑Liver glycogen content, ↑GS protein levels, ↓G6Pase activity	Govindarajan et al. (2021)
*Alsophila firma* (Baker) D.S.Conant [Cyatheaceae]/Mexican	Rhizome/Aqueous	Full extract	*In vitro:* G6Pase inhibition assay/IC_50_ = 341 μg/ml *In vitro:* FBPase inhibition assay/IC_50_ = 45 μg/ml	Andrade-Cetto et al. (2021b)
*Aster spathulifolius* Maxim. [Asteraceae]/Korean	Whole plant/EtOH	Full extract	*In vivo: db/db* mice (50, 100, and 200 mg/kg b.w.)/↑GK, ↓G6Pase and PEPCK expression	Yin et al. (2015)
*Averrhoa bilimbi* L. [Oxalidaceae]/Indian	Fruits/Aqueous	EtOAc fraction	*In vivo:* diabetic rats (25 mg/kg b.w.)/↓G6Pase and FBPase activity	Kurup and S, (2017)
*Bromelia karatas* L. [Bromeliaceae]/Mexican	Aerial parts/Aqueous	Full extract	*In vitro:* G6Pase inhibition assay/IC_50_ = 1,136 μg/ml	Mata-Torres et al. (2020)
*Calea urticifolia* (Mill.) DC. [Asteraceae]/Mexican	Aerial parts/Aqueous	Full extract	*In vivo:* diabetic rats (41 mg/kg b.w.)/↓Glucose production in PTTs *In vitro:* G6Pase inhibition assay/IC_50_ = 406 μg/ml	Andrade-Cetto et al. (2021a)
*Calotropis procera* (Aiton) W.T.Aiton [Apocynaceae]/Indian	Aerial parts/Latex	Protein fraction	*In vivo:* Wistar rats (5 mg/kg b.w.)/↑ pAMPK, ↓PEPCK expression, ↓blood glucose in PTTs	de Oliveira et al. (2019)
*Caralluma fimbriata* Wall. [Apocynaceae]/Indian	Stems/EtOH	Full extract	*In vivo:* insulin-resistant rats (200 mg/kg b.w.)/↓G6Pase and FBPase activity	Gujjala et al. (2017)
*Caralluma quadrangula* (Forssk.) N.E.Br./Apocynaceae/Saudi	Whole plant/MeOH	Russelioside B	*In vivo:* diabetic rats (50 mg/kg b.w.)/↑Glycogen content, ↓GP activity, ↓GS and GSK3β expression, ↓G6Pase activity and expression	Abdel-Sattar et al. (2016)
*Chrysobalanus icaco* L. [Chrysobalanaceae]/Nigerian	Leaves/Aqueous	Full extract	*In vivo:* diabetic rats (11.076, 22.134, and 44.268 mg/kg b.w.)/↑Liver glycogen content, ↓G6Pase activity	Ekakitie et al. (2021)
*Cola nitida* (Vent.) Schott & Endl. [Malvaceae]/African	Seeds/Aqueous	Full extract	*In vivo*: diabetic rats (300 mg/kg b.w.)/↓GP, G6Pase, and FBPase activities	Erukainure et al. (2019b)
*Combretum lanceolatum* Pohl ex Eichler [Combretaceae]/Brazilian	Flowers/EtOH	Full extract	*In vivo:* diabetic rats (500 mg/kg b.w.)/↑pAMPK and pAkt, ↓PEPCK expression	Siqueira et al. (2016)
*Coreopsis tinctoria* Nutt. [Asteraceae]/Chinese and Portuguese	Flowers/EtOAc	Marein	*In vitro:* cell culture (insulin-resistant HepG2)/↑Glycogen content, ↓G6Pase and PEPCK expression and protein levels	Jiang et al. (2018a)
*Corispermum squarrosum* L. [Amaranthaceae]/Mongol	Whole plant/EtOH	Oligosaccharides	*In vivo: db/db* mice (380 and 750 mg/kg b.w.)/In liver: ↑pIRS2, ↑pAkt, ↑IRS2, PI3K, Akt, and IR expression and protein levels	Bao et al. (2020)
*Couroupita guianensis* Aubl. [Lecythidaceae]	Leaves/Aqueous	Full extract Gold nanoparticles	*In vivo:* diabetic rats (100 and 2.5 mg/kg b.w.)/↑Glycogen storage, ↓G6Pase activity and expression	Manimegalai et al. (2020)
*Edgeworthia gardneri* (Wall.) Meisn. [Thymelaeaceae]/Chinese	Flowers/Aqueous	Full extract	*In vitro:* cell culture (insulin-resistant HepG2)/↑Glucose uptake and consumption, ↑Glycogen content, ↓Gluconeogenesis, ↑pIR, ↑pIRS1, ↑pAkt, ↑pGSK3	Zhang et al. (2020)
*Equisetum myriochaetum* Schltdl. & Cham. [Equisetaceae]/Mexican	Aerial parts/Aqueous	Full Extract	*In vivo:* diabetic rats (330 mg/kg b.w.)/↓Glucose production in PTTs	Mata-Torres et al. (2020)
*Eryngium cymosum* F.Delaroche [Apiaceae]/Mexican	Aerial parts/Aqueous	Full extract	*In vivo:* diabetic rats (470 mg/kg b.w.)/↓ Glucose production in PTTs *In vitro:* G6Pase inhibition assay/IC_50_ = 782 μg/ml *In vitro:* FBPase inhibition assay/IC_50_ = 57.4 μg/ml	Espinoza-Hernández et al. (2021)
*Eryngium longifolium* Cav. [Apiaceae]/Mexican	Aerial parts/EtOH	Full extract	*In vitro:* G6Pase inhibition assay/IC_50_ = 780 μg/ml *In vitro:* FBPase inhibition assay/IC_50_ = 93 μg/ml	Andrade-Cetto et al. (2021b)
*Ficus carica* L. [Moraceae]/Spain	Leaves/EtOH	Full extract	*In vivo:* diabetic mice (2 g/kg b.w.)/↓PEPCK and G6Pase expression, ↑pAMPK *In vitro:* cell culture (HepG2)/↓PEPCK and G6Pase expression, ↑pAMPK	Zhang et al. (2019d)
*Forsythia suspensa* (Thunb.) Vahl [Oleaceae]/Chinese	Fruit/MeOH	Full extract	*In vivo:* diabetic mice (200 mg/kg b.w.)/↓PEPCK expression	Zhang et al. (2016b)
*Graptopetalum paraguayense* (N.E.Br.) E.Walther [Crassulaceae]/Taiwan	Leaves/MeOH	Full extract Partially purified fraction (HH-F3)	*In vitro:* cell culture (Hep3B/T2)/↓PEPCK and G6Pase expression	Jhuang et al. (2015)
*Hyoscyamus albus* L. [Solanaceae]/Mediterranean	Seeds/MeOH	Calystegine fraction	*In vitro:* cell culture (insulin-resistant HepG2)/↑Glucose consumption, ↓G6Pase (catalytic subunit) expression, ↑IR, IRS1/2, PI3K, Akt1/2 expression and protein levels	Kowalczuk et al. (2021)
*Hypericum attenuatum* Fisch. ex Choisy [Hypericaceae]/Chinese	Whole plant/EtOH	Full extract	*In vivo:* insulin-resistant mice (100, 200, and 300 mg/kg b.w.)/↓PEPCK and G6Pase expression and protein levels, ↑GS expression and protein levels, ↑pIRS, ↑PI3K, ↑pAkt, ↑GSK3	Jin et al. (2019)
*Iris domestica* (L.) Goldblatt & Mabb. [Iridaceae]/Chinese	Leaves/EtOH	Saponins and polysaccharide fraction Flavonoid fraction	*In vivo:* KK-A^y^-mice (200 mg/kg b.w.)/↓G6Pase and PEPCK activities, ↑Glycogen content	Guo et al. (2019)
*Launaea acanthodes* (Boiss.) Kuntze [Asteraceae]/Iran	Aerial parts/EtOH	Full extract	*In vivo:* diabetic rats (100, 200, and 400 mg/kg b.w.)/↑GK and GLUT2 expression, ↓PEPCK and G6Pase expression	Marvibaigi et al. (2021)
*Lithocarpus polystachyus* (Wall. ex A.DC.) Rehder [Fagaceae]/Chinese	Leaves/Aqueous	Full extract	*In vivo:* insulin-resistant mice (800 mg/kg b.w.)/↑Glycogen content, ↑Liver glucose influx,/↓G6Pase and PEPCK expression, ↑IR and IRS expression	Wang et al. (2016a)
*Lupinus mutabilis* Sweet [Fabaceae]/Andean	Seeds/Aqueous	Protein fraction	*In vitro:* cell culture (HepG2)/↓glucose production and PEPCK expression	Muñoz et al. (2018)
*Myrianthus arboreus* P.Beauv. [Urticaceae]/African	Root bark/EtOH	EtOAc fraction Isoorientin Orientin Chlorogenic acid	*In vitro:* cell culture (H4IIE hepatocytes)/↓G6Pase activity, ↑pAMPK	Kasangana et al. (2019)
	Root bark/Aqueous, EtOH (EtOAc and hexane fractions), Alkaloid rich, and DCM extracts	Full extracts or fractions	*In vitro:* cell culture (H4IIE hepatocytes)/↓G6Pase activity, ↑pAkt, ↑pAMPK *In vitro:* cell culture (HepG2)/↑GS activity, ↑GSK3	Kasangana et al. (2018)
*Myrica rubra* (Lour.) Siebold & Zucc. [Myricaceae]/Chinese	Fruits/EtOH	Full extract	*In vivo:* KK-A^y^ mice (200 mg/kg b.w.)/↑pAMPK. ↓ PEPCK and G6Pase expression *In vitro:* cell culture (HepG2)/↑pAMPK, ↓PEPCK and G6Pase expression	Zhang et al. (2016a)
*Pachylobus edulis* G.Don [Burseraceae]/African and Nigerian	Leaves/EtOH	BuOH fraction	*In vivo:* diabetic rats (150 and 300 mg/kg b.w.)/↓GP, FBPase, and G6Pase activities	Erukainure et al. (2020)
	Leaves/EtOAc, EtOH, and Aqueous	Full extracts	*In vitro:* G6Pase inhibition assay/IC_50_ = 0.66, 3.59, 0.05 μg/ml	Erukainure et al. (2017)
*Panax ginseng* C.A.Mey. [Araliaceae]/Korean	Roots/EtOH	Black ginseng extract	*In vivo:* diabetic mice (300 and 900 mg/kg b.w.)/↓G6Pase, PEPCK and GP expression, ↑GS expression	Seo et al. (2016)
*Plantago depressa* Willd [Plantaginaceae]/Chinese	Seeds/EtOH	Plantadeprate A Plumbagine D Plantagoguanidinic acid	*In vitro:* cell culture (rat hepatocytes)/↓Gluconeogenesis inhibition by 8.2, 18.5, and 12.5% at 40 μM	Zheng et al. (2015)
*Raphia hookeri* G.Mann & H.Wendl. [Arecaceae]	Raffia palm wine/Concentrated in water bath	Concentrated wine	*In vivo:* diabetic rats (150 and 300 mg/kg b.w.)/↓GP, FBPase and G6Pase activity	Erukainure et al. (2019)
*Rhizophora mangle* L. [Rhizophoraceae]/Mexican	Bark/EtOH	Full Extract	*In vivo:* diabetic rats (90 mg/kg b.w.)/↓Glucose production in PTTs *In vitro:* G6Pase inhibition assay/IC_50_ = 99 μg/ml	Mata-Torres et al. (2020)
*Rhodiola crenulata* (Hook.f. & Thomson) H.Ohba [Crassulaceae]/Asian and Eastern European countries	Roots/EtOH	Full extract	*In vivo:* Sprague–Dawley rats (50 mg/kg b.w.)/↓PEPCK expression, ↑pAMPK *In vitro:* cell culture (HepG2)/↑pGSK3β and AMPK, ↓PEPCK and G6Pase expression	Lee et al. (2015)
*Sarcopoterium spinosum* (L.) Spach [Rosaceae]/Israel, Palestine, and Jordan	Root/Aqueous	Full extract	*In vivo:* insulin-resistant mice (35 and 100 mg/kg b.w.)/↑pIR, ↑pAkt, ↑GSK3, ↑glycogen content *In vivo:* KK-Ay mice (35 and 100 mg/kg b.w.)/↑pIR, ↑pAkt, ↓PEPCK expression	Rozenberg and Rosenzweig, (2018)
*Senna alata* (L.) Roxb. [Fabaceae]/Asia, Africa and South America	Leaves/EtOH	Full extract	*In vivo:* diabetic rats (400 mg/kg b.w.)/↑Liver glycogen content, ↑GS activity, ↓GP and FBPase activities	Mohanasundaram et al. (2021)
	Flowers/Aqueous	Full extract EtOAc fraction *n*-butanol fraction Aqueous fraction	*In vivo:* diabetic rats (75 mg/kg b.w.)/↑Glycogen storage	Uwazie et al. (2020)
*Sesbania grandiflora* (L.) Poir. [Fabaceae]/Aryuveda	Flowers/MeOH	Full extract	*In vivo:* diabetic rats (250 mg/kg b.w.)/↑Liver glycogen content, ↑GS activity, ↓GP, G6Pase, and FBPase activities	Sureka et al. (2021)
*Shirakiopsis elliptica* (Hochst.) Esser [Euphorbiaceae]/Nigerian	Leaves/EtOH	Full extract	*In vivo:* diabetic rats (400 and 800 mg/kg b.w.)/↑GK activity by 40.31%, ↓G6Pase activity by 37.29%, ↑Glycogen content	Ighodaro et al. (2017)
*Smilax moranensis* M.Martens & Galeotti [Smilacaceae]/Mexican	Roots/EtOH	Full Extract	*In vitro:* G6Pase inhibition assay/IC_50_ = 84 μg/ml	Mata-Torres et al. (2020)
*Swietena humilis* Zucc. [Meliaceae]/Mexican	Seeds/Aqueous	Dried aqueous extract Mexicanolide 1 Mexicanolide 2 Mexicanolide 3	*In vitro:* G6Pase inhibition assay in H4IIE hepatocytes Extract: 100 μg/ml = 40.67%; 200 μg/ml = 61.11 1: 9.46 µM = 42.68% 2: 8.79 µM = 56.51% 3: 8.13 µM = 41.79%	Ovalle-Magallanes et al. (2019)
*Tephrosia tinctoria* (L.) Pers. [Fabaceae]/Aryuveda	Stems/EtOAc	EtOAc fraction	*In vivo:* diabetic rats (100 and 200 mg/kg b.w.)/↑Liver glycogen content, ↓G6Pase and FBPase activity	Krishnasamy and Periyasamy, (2019)
*Terminalia catappa* L. [Combretaceae]/Aryuveda	Leaves/EtOH	Full extract	*In vivo:* diabetic rats (300 and 500 mg/kg b.w.)/↓G6Pase and FBPase activity	Divya et al. (2019)
*Trigonella foenum-graecum* L. [Fabaceae]/Asia, Africa, and the Mediterranean region	Seeds/EtOH	Fenugreek flavonoids	*In vivo:* diabetic rats (0.5 g in 10 ml/kg)/↑Liver glycogen content, ↓G6Pase and FBPase activity	Jiang et al. (2018b)

EtOH: ethanolic extract; MeOH: methanolic extract; EtOAc; ethyl acetate extract; PTT: pyruvate tolerance test; IR: insulin receptor; IRS1: insulin receptor substrate 1; IRS2: insulin receptor substrate 2; GSK3: glycogen synthase kinase 3; Akt: protein kinase B; PI3K: phosphoinositide 3-kinase; PEPCK: phosphoenolpyruvate carboxykinase; FBPase: fructose 1,6-bisphosphatase; G6Pase: glucose 6-phosphatase; GLUT4: glucose transporter 4; AMPK: AMP-activated protein kinase; GS: glycogen synthase; GP: glycogen phosphorylase.

## Discussion

Insulin resistance in liver leads to the release of large amounts of glucose into the bloodstream that affects long-term homeostasis. The regulation of hepatic glucose output represents a good pharmacological target for the control of metabolic diseases such as T2D, which are characterized by the presence of this pathophysiological phenomenon. The search for new molecules capable of regulating hepatic glucose metabolism from medicinal plants has focused on screening for phytochemicals that can directly inhibit key enzymes in glucose-producing pathways. However, considering compounds with the ability to also decrease the activity of the enzymes involved in terminating the insulin signal could result in more effective glycemic control.

According to the bibliographic search, plants used in different systems of traditional medicine have shown the ability to inhibit the activity or expression of PTP-1B, which could indicate that they have a potential inhibitory effect on HGP. The determination of biological activity of full extracts and compounds isolated from medicinal plants has been approached through different perspectives. Generally, the medicinal plant is first identified using an ethnopharmacological approach. Afterwards, different types of extracts are elaborated (aqueous, ethanolic, methanolic, etc.) and then tested on the biological activity to be evaluated following several paths: 1) direct inhibition enzymatic assays, which can be complemented with structure-activity relationship (SAR) studies and molecular docking analysis to find the possible structures responsible for the bioactivity, relating them with the binding of amino acid residues present at the catalytic or regulatory sites (regarding isolated compounds); 2) the use of cell cultures to evaluate the effect of the extract or compound on the expression and protein levels of key enzymes; and 3) *in vivo* studies, where diabetic (hyperglycemic) animals induced with STZ or alloxan, or insulin-resistant animals generated by the consumption of high-fat diet are used.

Regarding PTP-1B, most of the studies published between 2015 and 2021 focused on conducting enzyme activity assays, and few of them had a multidisciplinary approach that encompassed enzyme assays and *in vitro* or *in vivo* studies. The main problem with the first type of studies is that, although the inhibition potency and selectivity of the molecule over the enzyme are directly evaluated, the pharmacokinetic properties of the compound are omitted. This particularity stands out since it has been reported that, despite having excellent inhibitory activity, many compounds lack adequate cellular permeability, namely they present poor absorption and low bioavailability (Zhang et al., 2017). Another aspect to highlight is that PTP-1B is almost identical to TC-PTP, another member of the PTP family with 74% identity at the catalytic site, so it is important that the identified inhibitors have a high selectivity towards PTP-1B to avoid unwanted effects (Dewang et al., 2005). Considering these facts, it would be necessary in the future to carry out more studies involving as many approaches as possible to obtain a more integrative panorama and to be able to evaluate potential inhibitors considering their pharmacokinetic properties and selectivity. Also, it is encouraged to directly evaluate the effect of medicinal plants and their compounds with reported PTP-1B inhibitory capacity on hepatic glucose metabolism.

In addition to exhibiting PTP-1B inhibitory capacity, some of the medicinal plants reported in Table 1 also improved hepatic glucose metabolism by promoting glucose consumption and glycogen synthesis, upregulating activity or expression of GS, decreasing activity or expression of key enzymes involved in glycogenolysis and gluconeogenesis such as GSK3, GP, PEPCK, FBPase, and G6Pase, and by modulating insulin signaling. The compounds isolated from these plants could have a greater modulatory capacity of hepatic glucose metabolism because they are capable of directly reducing both insulin resistance and glucose production. These species were *Astragalus mongholicus* (astragaloside IV), *Chaenomeles japonica*, *Duranta erecta*, *Eriobotrya japonica* (maslinic acid, corosolic acid, oleanolic acid, and ursolic acid), *Symplocos cochinchinensis*, *Thonningia sanguinea* (2′-O- (3-O-galloyl-4,6-O-Sa-hexahydroxydiphenoyl-β-d-glucopyranosyl)-3-hydroxyphloretin, 4′-O-(4,6-O-Sa-hexahydroxydiphenoyl-β-d-glucopyranosyl)- phloretin, 2′-O-(3-O-galloyl-4,6-O-Sa-hexahydroxydiphenoyl-β-d-glucopyranosyl)phloretin, thonningianin A, and thonningianin B), *Vaccinium uliginosum* (cyanidin-3-arabinoside, delphinidin-3-glucoside, cyanidin-3-galactoside, cyanidin-3-glucoside, malvidin-3-galactoside, petunidin-3-glucoside, procyanidin B1, and procyanidin B2), and *Vigna radiata*. On the other hand, since *Coreopsis tinctoria*, *Lithocarpus polystachyus*, and *Panax ginseng* were documented in both Tables 1, 2, their isolated compounds may have better glycemic control.

This work focused on summarizing the medicinal plants with the potential capacity to reduce hyperglycemia resulting from an imbalance in the hepatic metabolism of glucose, encompassing two different approaches: the inhibition of PTP-1B (improvement of hepatic insulin resistance), and the modulation of enzymes involved in gluconeogenesis and glycogenolysis/glycogenesis (decreased hepatic glucose output). In recent years, PTP-1B research has focused on the characterization of different phytochemicals from medicinal plants, such as phenolic compounds, terpenes, and alkaloids. The main methodology used was to carry out direct enzyme inhibition tests to evaluate the potency of these molecules, omitting important aspects such as selectivity or pharmacokinetics. Therefore, it is proposed to use of multidisciplinary approaches that involve *in vitro* studies, such as the use of cell lines or primary culture to evaluate the effect of the extracts and compounds on expression and protein levels, and *in vivo* studies, where the concentration of the compound in systemic circulation and its duration is determined, as well as the transformation processes involved. In this regard, not only the inhibitory activity of the compounds is evaluated, but also the impact on other pharmacological aspects that can only be observed using animal models.

On the other hand, research on medicinal plants that modulate hepatic glucose metabolism has primary focused on testing full extracts rather than compounds. However, it is worth mentioning that mixtures could have synergistic effects capable of regulating multiple targets (Caesar and Cech, 2019) and therefore compound fractions may exhibit more bioactivity than isolated molecules. Further studies are needed to identify potential multi-target phytochemicals in plants listed in Table 2. Finally, it is expected that this review will provide greater knowledge of medicinal plants and compounds for the development of drugs that improving hepatic glucose metabolism as a therapeutic target for the treatment of T2D.

We suggest that *Coreopsis tinctoria*, *Lithocarpus polystachyus*, and *Panax ginseng* can be good candidates for developing herbal medicines or phytomedicines that target inhibition of hepatic glucose output as they can modulate the activity of PTP-1B, the expression of gluconeogenic enzymes, and the glycogen content. However, only their full extracts are tested until now. Therefore, compounds responsible for the effects mentioned above have not been identified, and pharmacological and toxicological tests in animal models are required to assess their efficacy and safety, with the aim of moving forward to carry out clinical studies.
